# Inhibiting Drivers of Non-mutational Drug Tolerance Is a Salvage Strategy for Targeted Melanoma Therapy

**DOI:** 10.1016/j.ccell.2016.02.003

**Published:** 2016-03-14

**Authors:** Michael P. Smith, Holly Brunton, Emily J. Rowling, Jennifer Ferguson, Imanol Arozarena, Zsofia Miskolczi, Jessica L. Lee, Maria R. Girotti, Richard Marais, Mitchell P. Levesque, Reinhard Dummer, Dennie T. Frederick, Keith T. Flaherty, Zachary A. Cooper, Jennifer A. Wargo, Claudia Wellbrock

**Affiliations:** 1 Manchester Cancer Research Centre, Wellcome Trust Centre for Cell-Matrix Research, The University of Manchester, Michael Smith Building, Oxford Road, Manchester, M13 9PT, UK; 2 Molecular Oncology Group, CRUK Manchester Institute for Cancer Research, Manchester Cancer Research Centre, Wilmslow Road, Manchester, M20 4BX, UK; 3 Department of Dermatology, UniversitätsSpital Zürich, University of Zürich, Gloriastrasse 31, 8091 Zurich, Switzerland; 4 Department of Medicine, Massachusetts General Hospital Cancer Center, 55 Fruit Street, Boston, MA 02114-2696, USA; 5 Divison of Surgical Oncology, University of Texas MD Anderson Cancer Center, 1400 Pressler Street, Houston, TX 77030, USA

## Abstract

Once melanomas have progressed with acquired resistance to mitogen-activated protein kinase (MAPK)-targeted therapy, mutational heterogeneity presents a major challenge. We therefore examined the therapy phase before acquired resistance had developed and discovered the melanoma survival oncogene *MITF* as a driver of an early non-mutational and reversible drug-tolerance state, which is induced by PAX3-mediated upregulation of MITF. A drug-repositioning screen identified the HIV1-protease inhibitor nelfinavir as potent suppressor of PAX3 and MITF expression. Nelfinavir profoundly sensitizes *BRAF* and *NRAS* mutant melanoma cells to MAPK-pathway inhibitors. Moreover, nelfinavir is effective in *BRAF* and *NRAS* mutant melanoma cells isolated from patients progressed on MAPK inhibitor (MAPKi) therapy and in *BRAF/NRAS/PTEN* mutant tumors. We demonstrate that inhibiting a driver of MAPKi-induced drug tolerance could improve current approaches of targeted melanoma therapy.

## Highlights

• MITF is a driver of a reversible non-mutational drug-tolerance phase in melanoma
• Drug repositioning identifies nelfinavir mesylate as a suppressor of MITF expression
• Nelfinavir sensitizes *BRAF* and *NRAS* mutant melanoma to MAPK inhibitor treatment
• A nelfinavir combination therapy overcomes *NRAS*-driven acquired resistance

## Graphical Abstract

**Figure:**
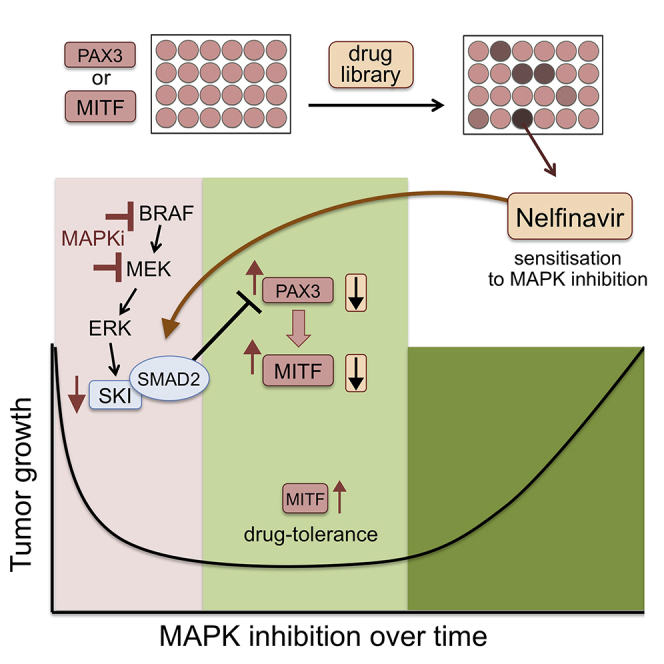

## Teaser

Smith et al. discover PAX3-mediated overexpression of *MITF* as a reversible resistance mechanism to MAPK-pathway inhibition in *BRAF* mutant melanomas and identify nelfinavir, which inhibits this mechanism and sensitizes not only BRAF mutant but also *BRAF* and *NRAS* mutant melanoma cells to MAPK-pathway inhibitors.

## Significance

**The immense genetic heterogeneity found in mutational acquired resistance to targeted therapy highlights the need for more effective treatment before resistance occurs. By focusing on melanomas during the initial response phase of treatment, we discovered that the upregulation of the melanoma survival oncogene *MITF* drives early drug tolerance. This process is reversible; revealing the non-mutational nature of the MITF-mediated drug tolerance. Importantly, we demonstrate that this non-mutational tolerance phase, which precedes acquired mutational resistance, provides an opportunity for more effective treatment approaches. By repositioning an HIV drug to target MITF as a driver of MAPK inhibitor (MAPKi)-induced drug tolerance we identify a clinically relevant approach for melanoma therapy that has the potential to improve initial responses and delay the onset of resistance.**

## Introduction

The identification of the vast genetic heterogeneity in tumors of cancer patients progressed on targeted therapy (Burrell et al., 2013) reveals a major challenge and emphasizes the need to improve effectiveness of treatment before mutational acquired resistance prevails. Clearly, there is room for improvement and in melanoma this is highlighted by the observed increase in progression-free survival in BRAF/MEK inhibitor combination therapies compared with BRAF inhibitor monotherapies (Larkin et al., 2014, Long et al., 2015).

In *BRAF* mutant melanoma cells, BRAF is the driver of cellular signaling — the prerequisite to BRAF-targeted therapy (Salama and Flaherty, 2013). Moreover, in a patient who shows a significant response to BRAF inhibitors, BRAF-driven cells must be dominating the tumor(s) at the time of treatment when the drug affects the majority of cells. This is crucial, because our knowledge about mitogen-activated protein kinase (MAPK)-signaling networks (Lito et al., 2012, von Kriegsheim et al., 2009) suggests that, in the initial phase of inhibitor treatment, a fairly uniform response will occur while the BRAF-driven signaling network readjusts. This readjustment will allow a cell to quickly adapt to the new input. Importantly, it is this uniform response to MAPK-pathway inhibition that we might be able to take advantage of. If the driver of this newly established fitness could be targeted before heterogeneity of acquired resistance develops, this should significantly prolong responses and hence delay the occurrence of acquired resistance.

Surprisingly, while enormous effort has gone into understanding the molecular events in mutational acquired resistance, not much attention has been given to what happens during treatment, particularly during the early phase when patients still respond to drug treatment with inhibition of the MAPK pathway. BRAF inhibitor-induced rewiring can occur within the first 24 hr leading to a dampening of the inhibitor effect (Lito et al., 2012). Other adaptive signaling seen in melanoma cells within 24–48 hr involves an altered oxidative metabolism (Haq et al., 2013a), increased phosphorylation of AKT (Gopal et al., 2010), and upregulation of ERBB3 (Abel et al., 2013). Exposure to MAPK inhibitor (MAPKi) for 9–12 days can enrich drug-tolerant melanoma cell populations that display chromatin modifications paralleled by upregulation of histone demethylases (Menon et al., 2015, Sharma et al., 2010). Selection for sub-populations might also occur as seen with epidermal growth factor receptor (EGFR)-expressing cells (Sun et al., 2014). Nevertheless, EGFR, ERBB3, and AKT also display increased expression and/or phosphorylation in the majority of progressed melanomas (Abel et al., 2013, Girotti et al., 2013, Long et al., 2014). This suggests that the above-described events are not reversible when the MAPK pathway becomes re-activated.

We and others have previously reported that the melanoma transcription factor MITF can provide resistance to MAPK-pathway inhibitors through various mechanisms, such as enhancing survival signaling and altering metabolism (Gopal et al., 2014, Haq et al., 2013a, Haq et al., 2013b, Johannessen et al., 2013, Smith et al., 2013, Wellbrock and Arozarena, 2015). Enhanced MITF expression is linked to innate resistance, and increased MITF expression as well as *MITF* amplification is found in some progressed melanomas (Gopal et al., 2014, Ji et al., 2015, Muller et al., 2014, Van Allen et al., 2014). Importantly, not only are *MITF* focal amplifications significantly linked to the *BRAF* mutant melanoma subtype (Cancer Genome Atlas Network, 2015), but the expression of MITF is also tightly regulated by BRAF-initiated MAPK signaling (Wellbrock and Marais, 2005, Wellbrock et al., 2008). This led us to investigate its potential involvement in driving increased fitness during the initial phases of treatment.

## Results

### MITF Expression Is Upregulated in Response to Long-Term BRAF and MEK Inhibition

**Figure 1:**
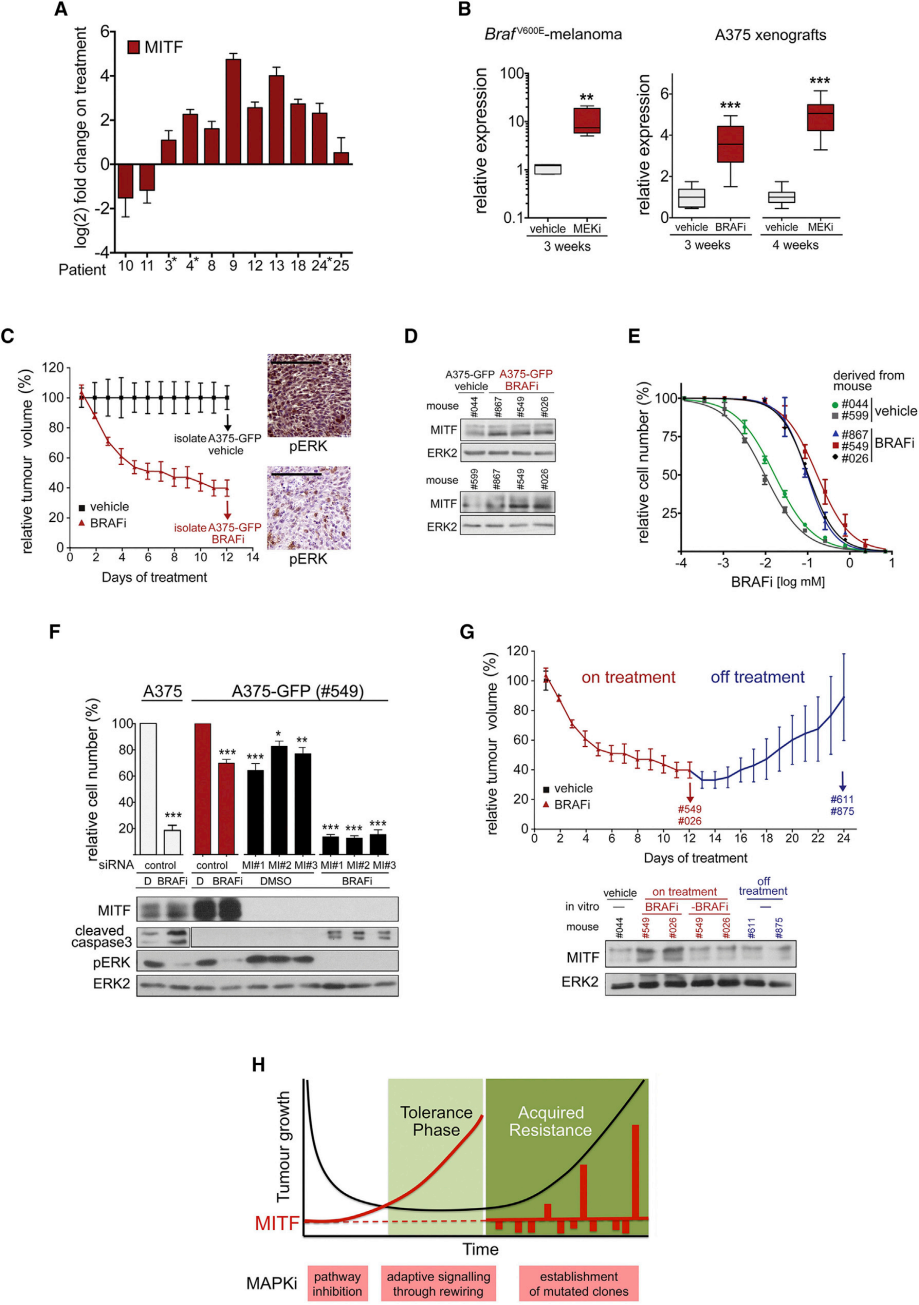
MITF Expression Is Upregulated in Response to Long-Term BRAF and MEK Inhibition (A) qRT-PCR for MITF expression (mean ± SD) in melanoma of patients undergoing treatment with vemurafenib (^∗^) or a dabrafenib/trametinib combination. (B) qRT-PCR for MITF expression in *Braf*^V600E^ murine melanoma allografts from mice treated with vehicle or 25 mg/kg once daily (qd) PD184352 (MEKi) (each group: n = 5), and in A375 xenografts from mice treated with vehicle, 10 mg/kg qd selumetinib (MEKi) or 25 mg/kg qd PLX4720 (BRAFi) (each group: n = 3). Data show box plots indicating the upper/lower quartile and the median with whiskers from min to max values. (C) A375-GFP cells were isolated from xenografts grown in mice treated with vehicle (n = 4) or 100 mg/kg qd vemurafenib (BRAFi, n = 7) for 12 days. Mean relative tumor volume ± SEM and a phospho-ERK immunohistochemistry are shown; scale bars, 200 μm. (D) Western blot for MITF and ERK2 in A375-GFP cells after isolation from the indicated mice. (E) Dose-response curve (mean ± SEM) of A375-GFP cells treated with vemurafenib (BRAFi) for 48 hr. (F) A375-GFP cells isolated from a vemurafenib (BRAFi)-treated mouse were transfected with control or MITF-specific siRNAs and left in DMSO or cultured in the presence of vemurafenib (BRAFi) for 72 hr. Naive A375 cells were used as control. Relative cell numbers (mean ± SEM) and western blots are shown. (G) After treatment with vemurafenib (BRAFi) for 12 days as described in (C), three mice were kept off drug for another 12 days, before A375-GFP cells were isolated from xenografts (#611, #875). Mean relative tumor volume ± SEM and a western blot for MITF and ERK2 are shown. In parallel, A375-GFP cells isolated on treatment (#549, #026) were maintained with vemurafenib (BRAFi) or without drug (-BRAFi) for 12 days and analyzed for MITF. (H) Model describing a phase of non-mutational drug tolerance during which MAPK-pathway inhibition triggers adaptive signaling. For (B) and (F): ^∗^p < 0.05; ^∗∗^p < 0.01; ^∗∗∗^p < 0.001. See also Figure S1 and Table S1.

We analyzed melanomas from 11 patients undergoing treatment with vemurafenib or a dabrafenib/trametinib combination (Table S1), and found that, within the first 2 weeks of treatment, MITF expression was upregulated in 9 of 11 patients (Figure 1A). In all samples, expression of the ERK target *DUSP6* was decreased (Figure S1A), indicating that the MAPK pathway was inhibited, albeit to different degrees. The upregulation of MITF correlated with increased expression of its target genes *TYR* and *MLANA* (Figure S1A), which is in line with previous observations of increased melanoma differentiation antigen expression on treatment (Frederick et al., 2013), and indicates that MITF is functional. We also observed MITF and target gene upregulation in *Braf*^V600E^ mouse melanoma allografts in syngeneic mice treated with an MEK inhibitor, as well as in human *BRAF*^V600E^ melanoma xenografts grown in mice treated with either a BRAF inhibitor or an MEK inhibitor, and again this correlated with downregulation of *DUSP6* expression (Figures 1B and S1B).

To analyze the consequences of this upregulation, we isolated A375-GFP melanoma cells from tumor-bearing mice treated with the BRAF inhibitor vemurafenib (100 mg/kg) for 12 days, at which point the tumor response was reaching a plateau (Figure 1C). Confirming our previous observations, the cells isolated from vemurafenib-treated tumors expressed increased MITF when compared with cells isolated from vehicle-treated tumors (Figure 1D). Importantly, the cells that had been exposed to the drug in the tumors of treated mice were more tolerant to BRAF inhibition than cells isolated from untreated tumors, with an over 10-fold increase in the concentration causing 50% of maximal growth inhibition (GI_50_) (Figure 1E). Moreover, in agreement with the previously described function of MITF in resistance to MAPK inhibitors (Haq et al., 2013b, Johannessen et al., 2013, Muller et al., 2014, Smith et al., 2013), depletion of MITF rendered drug-tolerant A375-GFP cells sensitive again (Figure 1F). Notably, after drug removal the upregulation of MITF seen in A375-GFP cells was reversible in vivo as well as in vitro (Figure 1G). The latter was also seen in A375-T cells, in vitro long-term MEK inhibitor-treated A375 cells, which are tolerant when on drug treatment, but become sensitive again when off treatment (Figures S1C–S1E). These observations are crucial as it demonstrates that in order for MITF to provide tolerance to MAPK inhibitors, no mutational event is required.

Our data suggest that a buildup of MITF expression occurs as a direct adaptive response to MAPKi treatment during a tolerance phase (Figure 1H). This phase of pathway inhibition and rewiring precedes acquired resistance, and, importantly, recent data suggest that during this phase, rewired cells can support the outgrowth of mutated clones with inherent resistance, thereby contributing to the establishment of acquired resistance (Obenauf et al., 2015). Indeed, drug-tolerant A375-GFP cells display increased expression of genes characteristic for rewired cells (Figure S1F). In our model (Figure 1H), mutated clones that display cell-autonomous resistance will eventually re-establish tumor growth. Many (mutated) drivers of acquired resistance, often leading to pathway reactivation and profound heterogeneity, have been identified. However, our data suggest that increased MITF is driving an early drug-tolerance phase.

### A Drug Screen to Target PAX3 and MITF as Potential Drivers of Early Drug Tolerance Identifies Nelfinavir

**Figure 2:**
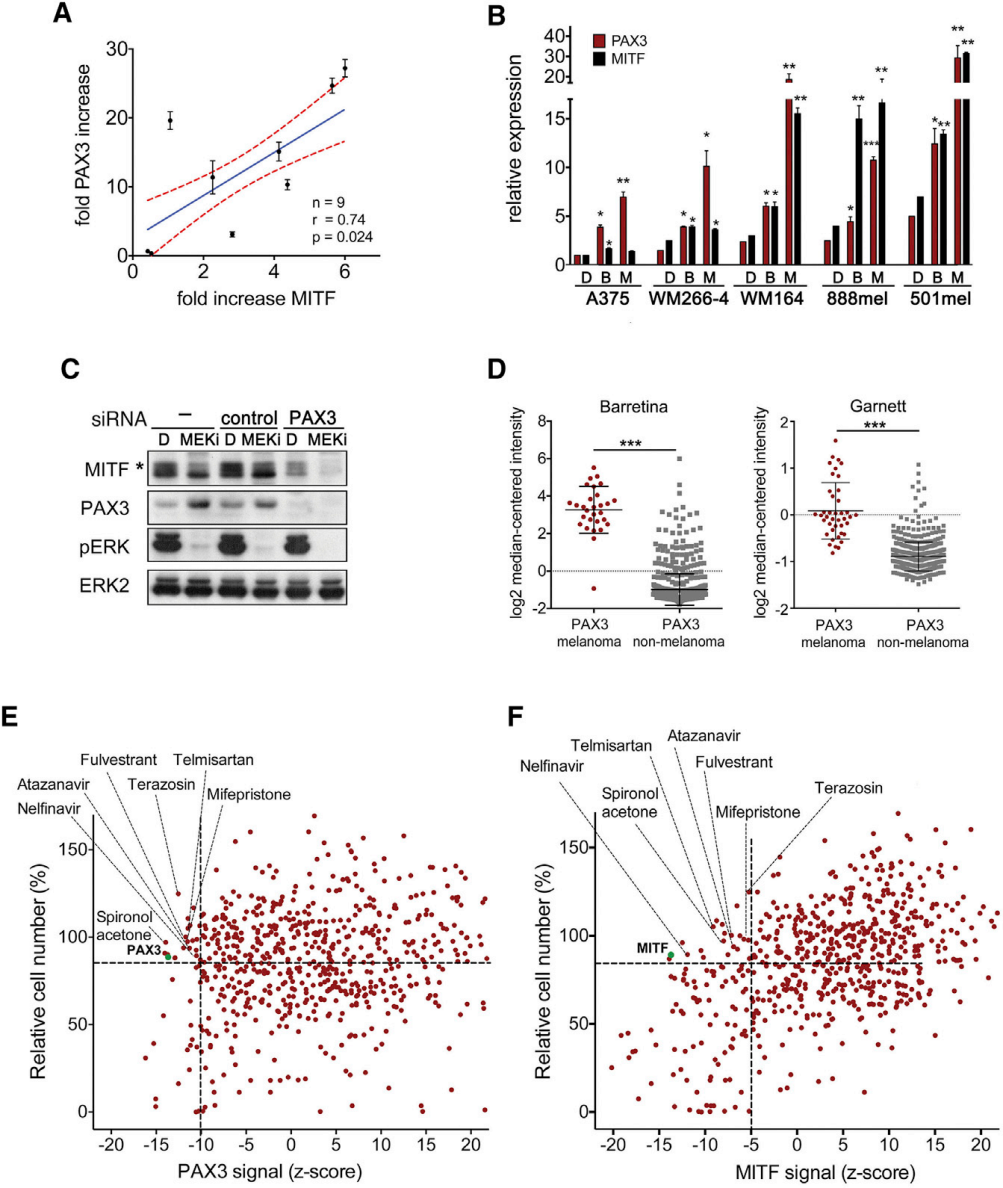
A Drug Screen to Target PAX3 and MITF (A) Correlation of fold change in MITF and PAX3 expression (mean ± SD) in melanomas of patients (n = 9) undergoing treatment with vemurafenib or dabrafenib/trametinib combination. Shown is the mean (blue) with the 95% confidence interval (red dashed line). (B) qRT-PCR analysis for PAX3 and MITF expression (mean ± SEM, ^∗^p < 0.05; ^∗∗^p < 0.01; ^∗∗∗^p < 0.001) in a panel of melanoma cell lines treated with DMSO, vemurafenib (B) or selumetinib (M) for 48 hr. (C) Western blot of WM266-4 cells untreated or treated with a control or PAX3-specific siRNA in the presence of DMSO or PD184352 (MEKi, 24 hr). The asterisk indicates an ERK-phosphorylated MITF form. (D) PAX3 expression analysis of the Barretina and Garnett datasets deposited in Oncomine. Data show scatter dot plots, indicating the mean ± SD. ^∗∗∗^p < 0.001. (E) WM266-4 cells treated with an FDA-approved drug library were analyzed for PAX3 expression and viability. Values are presented as % relative cell number and *Z* score. Drugs with a survival score >90% and a *Z* score <−10 were nominated candidate drugs. (F) Drug screen for MITF expression as described in (E). Values are presented as % relative cell number and *Z* score. Drugs with a survival score >90% and a *Z* score <−5 were nominated candidate drugs. See also Figure S2.

With the idea that targeting the MITF buildup would affect the tolerance phase and thus delay the onset of acquired resistance, we embarked on identifying the cause of MITF upregulation in response to MAPKi. We assessed crucial regulators of *MITF* in biopsies from patients on treatment, and found a significant correlation of *MITF* transcripts with the expression of the paired-box transcription factor PAX3 (Figure 2A). Thus, PAX3, a well-known transcriptional regulator of MITF (Kubic et al., 2008), is upregulated during MAPKi treatment, which is also seen within 48 hr in a panel of *BRAF* mutant melanoma cell lines (Figure 2B). The upregulation of PAX3 was paralleled by MITF, but its upregulation was only marginal during this time in cell lines expressing low basal MITF levels (A375, WM266-4) (Figure 2B). Nevertheless, MITF expression increases at later time points (see A375-T, Figure 1G) and this delay is due to a complex competitive regulation of the *MITF* promoter involving additional transcriptional regulators (Wellbrock et al., 2008). This delay is also seen at protein level in A735 cells, where depletion of PAX3 in the presence of MEK inhibitor strongly suppresses MITF expression (Figure 2C).

PAX3 expression is highly enriched in cutaneous melanoma compared with >170 other cancer types (Figure 2D). In addition, we have shown previously that reduced PAX3 expression sensitizes melanoma cells to MEK inhibitors (Smith et al., 2013). These findings make the PAX3-MITF axis a good target to counteract MITF-driven drug tolerance. We performed an immunofluorescence-based screen using a library of 640 US Food and Drug Administration (FDA)-approved drugs to identify compounds that will target PAX3 and MITF expression (Figure S2A). We also assessed melanoma cell survival in response to the drug library and set a threshold at the effect on survival induced by RNAi-mediated depletion of PAX3 or MITF, respectively (Figures 2E and 2F). For PAX3 expression we set a threshold of a *Z* score of −10, which led to the identification of 18 drugs resulting in significant downregulation of PAX3 expression. For MITF expression we set a threshold of a *Z* score of −5, because we wanted to account for delayed effects on MITF with it being a PAX3 target gene.

Applying these criteria, we identified seven drugs that reduced both PAX3 and MITF expression (Figures 2E and 2F, and S2B). Among these drugs nelfinavir mesylate, an HIV1-protease inhibitor that had shown anti-neoplastic activity (Chow et al., 2009), had the strongest effect on the expression of both PAX3 and MITF.

### PAX3 and MITF Suppression Is Required for Nelfinavir-Induced Growth Inhibition

**Figure 3:**
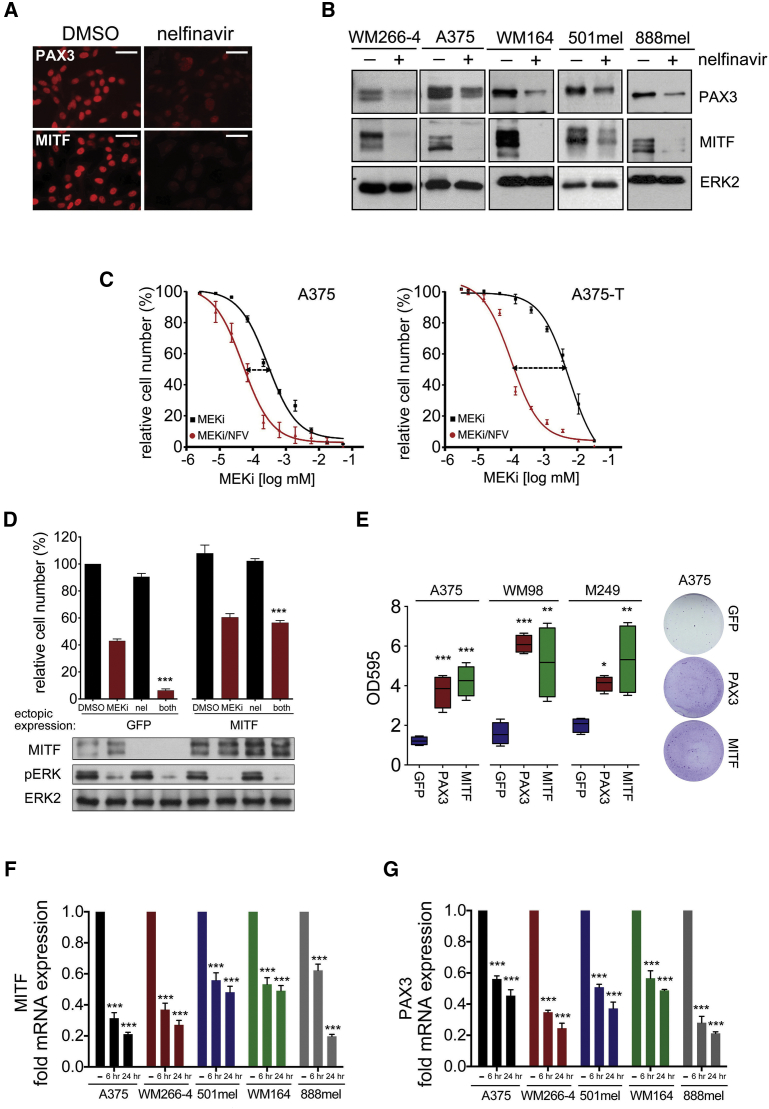
Nelfinavir Suppresses PAX3 and MITF Expression in Melanoma Cells (A) Immunofluorescence analysis for PAX3 and MITF in WM266-4 cells left untreated or treated with 10 μM nelfinavir for 24 hr; scale bars, 50 μm. (B) Western blot of the indicated cell lines treated with 10 μM nelfinavir for 24 hr for PAX3, MITF, and ERK2. (C) Dose-response curve (mean ± SEM) for A375 or A375-T cells treated with 7 μM nelfinavir for 24 hr followed by 48 hr selumetinib (MEKi) treatment. (D) Melanoma cells ectopically expressing GFP or MITF were treated with 10 μM nelfinavir and selumetinib (MEKi) alone or in combination for 72 hr before cell number analysis (mean ± SEM). A MITF, pERK, and ERK2 western blot is shown. (E) Colony survival analysis after 3 weeks of nelfinavir/selumetinib (MEKi) treatment using the indicated cell lines transfected with an empty vector or a PAX3- or MITF-expressing vector. Data show box plots indicating the upper/lower quartile and the median with whiskers from min to max values. (F) qRT-PCR analysis for MITF expression (mean ± SEM) in melanoma cell lines treated with DMSO or with 10 μM nelfinavir. (G) qRT-PCR analysis for PAX3 expression (mean ± SEM) in the samples used in (F). For all panels: ^∗^p < 0.05; ^∗∗^p < 0.01; ^∗∗∗^p < 0.001. See also Figure S3.

Nelfinavir efficiently suppressed PAX3 and MITF expression in a panel of *BRAF* mutant melanoma cells (Figures 3A and B) and reduced the GI_50_ for the MEK inhibitor selumetinib in drug-tolerant A375 melanoma cells (A375-T) by ∼60-fold, comparable with the GI_50_ in sensitive cells (Figure 3C). Moreover, the GI_50_ of nelfinavir correlates with PAX3 and MITF expression levels (Figures S3A), and ectopic overexpression of PAX3 or MITF rescued, whereas MITF depletion enhanced the growth inhibition induced by nelfinavir and MEK inhibition (Figures 3D and 3E, and S3B–S3D). Together, this indicates that suppression of PAX3 and MITF is contributing to the growth inhibitory effects.

We next aimed to identify how nelfinavir affects PAX3 and MITF expression. MITF mRNA levels were reduced within 24 hr of nelfinavir treatment (Figure 3F), suggesting that PAX3 regulates *MITF* transcription and is the nelfinavir target. To assess events upstream of PAX3, we analyzed phosphatidylinositol 3 (PI3)-kinase/AKT signaling and HSP90 activity as they can be targeted by nelfinavir (Gantt et al., 2013, Gills et al., 2007, Shim and Liu, 2014). However, we did not observe loss of AKT phosphorylation or changes in the HSP90 client protein AKT at times when reduced PAX3 expression occurred (Figure S3E). Furthermore, there was no effect on BRAF protein levels in cells expressing *BRAF*^V600E^ (Figure S3F), another HSP90 client protein (da Rocha Dias et al., 2005). These findings confirm previous data that nelfinavir does not target PI3-kinase signaling in melanoma cells (Jiang et al., 2007), and rules out an involvement of HSP90 in the inhibitory effect of nelfinavir on PAX3 protein levels. Moreover, nelfinavir affects PAX3 mRNA expression (Figure 3G), suggesting that the transcriptional regulation of PAX3 is suppressed by nelfinavir.

### Nelfinavir Suppresses PAX3 Expression through SMAD2/4 and SKI

**Figure 4:**
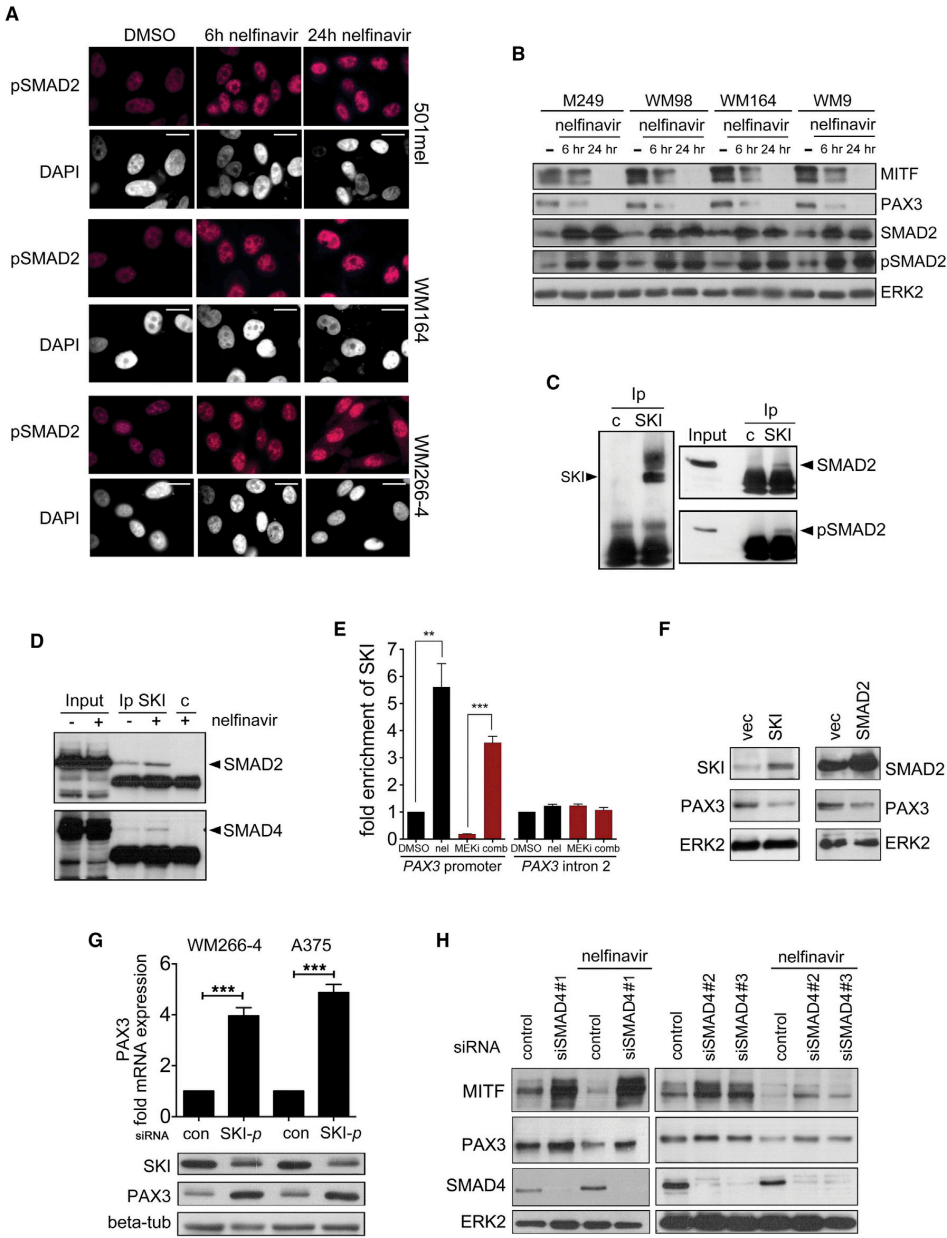
Nelfinavir Suppresses PAX3 through a SMAD2/SMAD4/SKI Complex (A) Immunofluorescence analysis for phospho-SMAD2 in melanoma cells treated with nelfinavir; scale bars, 10 μm. (B) Western blot for MITF, PAX3, SMAD2, pSMAD2, and ERK2 in melanoma cells treated with nelfinavir. (C) SKI immunoprecipitates from melanoma cells were analyzed for the presence of SKI, SMAD2, and pSMAD2. (D) SKI immunoprecipitates from untreated or nelfinavir (10 μM)-treated melanoma cells were analyzed for the presence of SMAD2 and SMAD4. (E) Chromatin immunoprecipitation analysis from A375 cells treated with selumetinib (MEKi) or nelfinavir (10 μM) for 24 hr alone or in combination using SKI antibodies or non-specific antibodies. The region of the PAX3 promoter spanning the SMAD binding site (−135/−98) was amplified. Relative binding in DMSO was set 1; shown are mean values ± SEM A region in the PAX3 intron2 was used as control. (F) Melanoma cells transfected with a vector control or an SKI or SMAD2 expression plasmid were analyzed for PAX3 by western blotting. (G) qRT-PCR and western blot for PAX3 in cells treated with control or SKI-specific siRNAs (using an SMART-pool [SKI-*p*] of four siRNAs). (H) Melanoma cells transfected with control or different SMAD4-specific siRNAs were left untreated or treated with nelfinavir for 24 hr and analyzed for indicated proteins by western blotting. All error bars are ± SEM from the mean. ^∗∗^p < 0.01; ^∗∗∗^p < 0.00. See also Figure S4.

In melanocytes, the transcriptional co-suppressor SKI regulates expression from the *PAX3* promoter. This is controlled by transforming growth factor β (TGF-β), which induces SMAD2 phosphorylation and the formation of a SMAD2/4/SKI repressor complex (Yang et al., 2008). Melanoma cells, however, often display constitutive activation of TGF-β signaling, and this is reflected in a steady-state presence of nuclear phospho-SMAD2 (Figure 4A). Nelfinavir increased the amount of SMAD2 and consequently nuclear phospho-SMAD2 in melanoma cell lines in the absence of exogenous TGF-β (Figures 4A and 4B), and, importantly, this correlated with the reduction in PAX3 and MITF expression (Figure 4B).

For SMAD2 to act as suppressor for *PAX3* it requires SKI (Xu et al., 2000, Yang et al., 2008), and we detected SMAD2 in SKI immunoprecipitates from melanoma cells under steady-state conditions (Figure 4C). Nelfinavir treatment increased the recruitment of not only SMAD2 but also SMAD4 to SKI (Figure 4D) and the recruitment of SKI to the *PAX3* promoter (Figure 4E). The suppressor function of SKI is maintained in melanoma, where its overexpression led to a reduction and its depletion to an increase in PAX3 and MITF expression (Figures 4F and 4G, S4A and S4B). Likewise the overexpression of SMAD2 suppressed PAX3 expression (Figures 4F and S4C), and while depletion of SMAD4 or SMAD2 increased PAX3 levels, nelfinavir was not able to effectively suppress PAX3 in the absence of the SMADs (Figures 4H and S4D and S4E).

### MEK Regulates the SKI-Mediated Suppression of PAX3

Because we had identified the SMAD2/4/SKI complex as relevant for the inhibitory action of nelfinavir on *PAX3* transcription, and nelfinavir counteracted the MAPKi-induced tolerance in melanoma cells (see Figure 3C), we wanted to identify the link between the SMAD/SKI suppressor complex and MAPK signaling.

**Figure 5:**
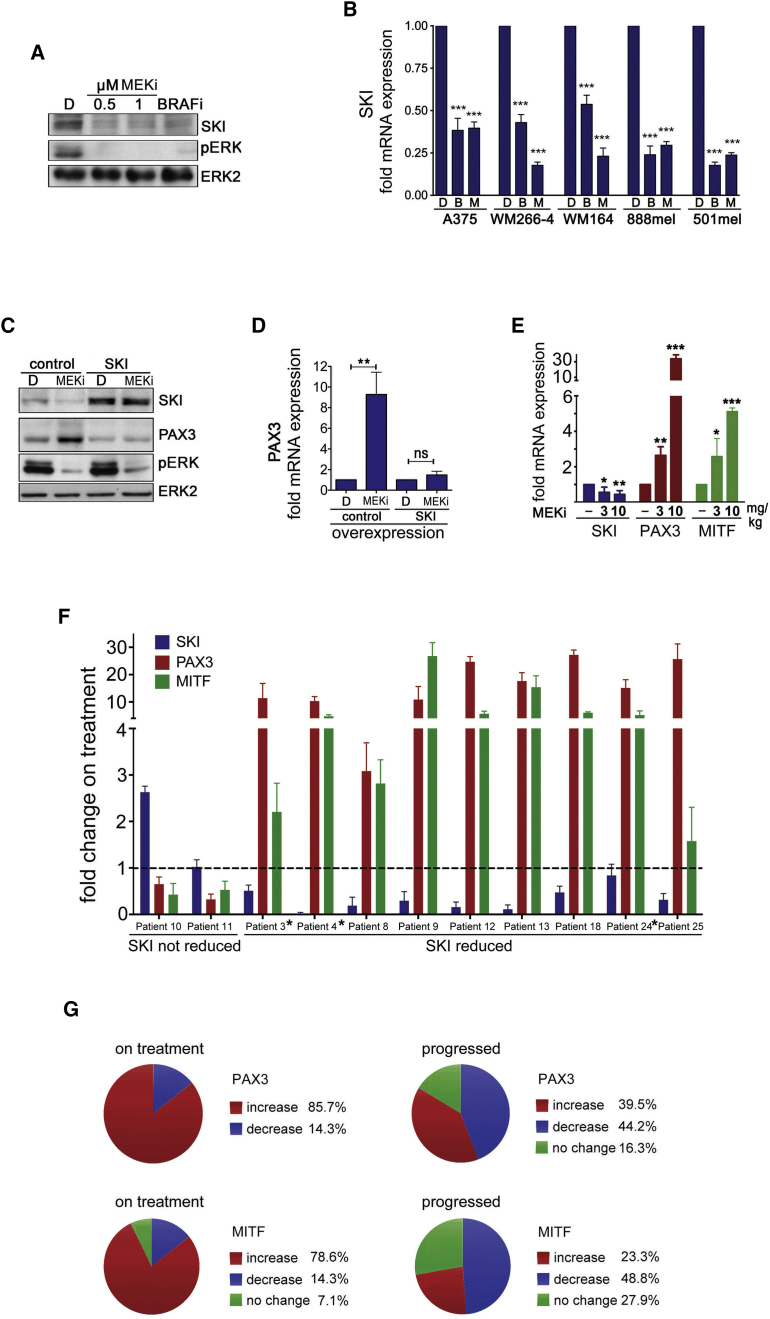
MEK Suppresses PAX3 through SKI (A) Western blot of WM266-4 cells treated for 24 hr with DMSO, PD184352 (MEKi), or vemurafenib (BRAFi) for SKI, pERK, and ERK2. (B) qRT-PCR for SKI expression (mean ± SEM) in the indicated melanoma cell lines treated with vemurafenib (BRAFi) or selumetinib (MEKi) for 48 hr. (C) Western blot of WM266-4 cells transfected with a control or SKI expression plasmid for SKI, PAX3, pERK, and ERK2. Cells were treated for 24 hr with DMSO or PD184352 (MEKi). (D) qRT-PCR analysis for PAX3 (mean ± SD) in WM266-4 treated as in (C). (E) qRT-PCR analysis for SKI, PAX3, and MITF expression (mean ± SEM) in A375 melanoma xenografts from mice treated with selumetinib (MEKi) for 4 weeks. (F) qRT-PCR analysis of SKI, PAX3, and MITF (mean ± SD) in patients on treatment (2 weeks) with vemurafenib (^∗^) or dabrafenib/trametinib inhibitor combination. (G) Analysis of publicly available gene expression datasets GEO: GSE50509 (21 patients) and GEO: GSE61992 (9 patients) as well as our dataset (11 patients) for fold changes in PAX3 and MITF expression. In total 41 pre-treatment, 14 on treatment, and 43 progressed samples were analyzed; % changes are indicated. For all graphs: ^∗^p < 0.05; ^∗∗^p < 0.01; ^∗∗∗^p < 0.001. See also Figure S5.

ERK can regulate SMAD function at various levels in TGF-β signaling, but we did not detect any effect of MEK inhibition on SMAD2 steady state or TGF-β-induced nuclear localization, or TGF-β-stimulated transcription of *SPARC* or *VEGF* in melanoma cells (Figures S5A and S5B). However, MEK activity was relevant for the TGF-β-mediated suppression of *PAX3* (Figure S5B). This suggested a link between MEK and the transcriptional co-suppressor SKI, and indeed BRAF or MEK inhibition reduced SKI protein and mRNA levels in melanoma cells (Figures 5A and 5B). Furthermore, SKI overexpression from an ectopic promoter prevented the upregulation of PAX3 expression otherwise seen when the MAPK pathway is inhibited (Figures 5C and 5D). Similar results were found with SMAD2, whose overexpression also enhanced the growth inhibitory effect of MEK inhibition (Figures S5C and S5D). Thus, our data suggest a mechanism whereby BRAF and MEK stimulate the expression of SKI, which together with SMAD2 suppresses the *PAX3* promoter. However, inhibition of BRAF or MEK relieves the SKI suppressor activity and increases *PAX3* transcription, which will eventually increase MITF expression. In line with this, SKI recruitment to the *PAX3* promoter is reduced in the presence of an MEK inhibitor and this is counteracted by nelfinavir (see Figure 4E).

The individual functional links supporting such a mechanism were seen in vivo in A375 tumors, where reduced SKI expression correlated with increased PAX3 and MITF expression in a dose-dependent manner (Figure 5E). Moreover, we observed a similar correlation in patients on MAPKi treatment. SKI expression was reduced in nine patients and this was correlated with an upregulation of PAX3 and MITF expression (Figure 5F). However, in two patients SKI expression was not reduced and PAX3 and MITF expression dropped below the initial expression levels before treatment (Figure 5F).

In line with the idea that the observed PAX3/MITF response is a consequence of MAPK-pathway inhibition and as such occurs while patients are still responding to treatment, we found that in tumors from our cohort of patients whose melanoma had progressed, PAX3 and MITF expression were generally reduced and SKI expression was restored (Figures S5E and S5F). This correlated with the recovery of *DUSP6* expression and ERK phosphorylation in three available patient samples (Figure S5G), suggesting pathway reactivation in these tumors. A similar correlation was seen in gene expression datasets (Figure S5H) derived from two different patient cohorts (Long et al., 2014, Rizos et al., 2014). Analysis of these datasets further revealed increased PAX3 and MITF expression in ∼40% and ∼23% of progressed tumors, respectively (Figures S5I and S5J, and 5G). However, analysis of all “on treatment” datasets found that ∼80% of tumors display upregulated PAX3/MITF expression before progression (Figure 5G).

### Nelfinavir Sensitizes to BRAF and MEK Inhibition in *BRAF* Mutant Melanoma

**Figure 6:**
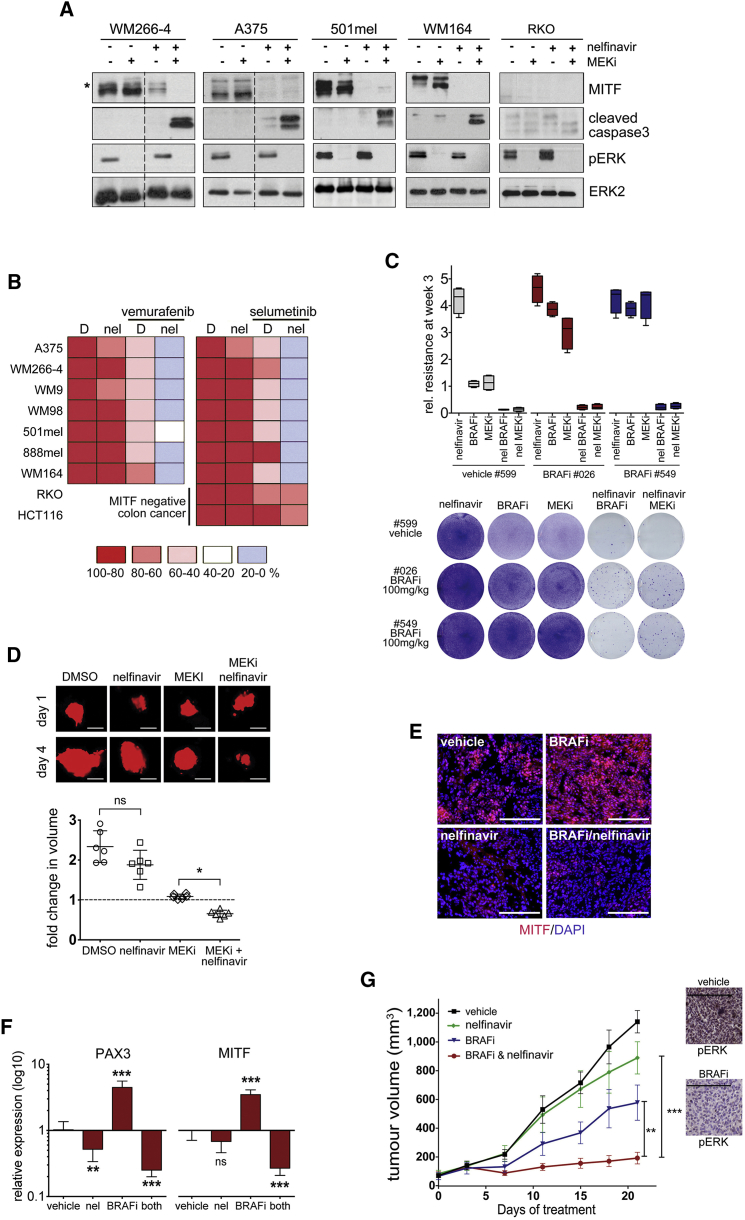
Nelfinavir Sensitizes *BRAF* Mutant Melanoma to MAPK Inhibition (A) Western blot of indicated cell lines incubated with nelfinavir (A375 7 μM, others 10 μM) 24 hr prior to a 48-hr treatment with DMSO or selumetinib (MEKi) for the indicated proteins. The asterisk indicates an ERK-phosphorylated form of MITF. (B) Indicated cells lines were treated with 10 μM nelfinavir 24 hr prior to treatment with DMSO, selumetinib (MEKi), or vemurafenib (BRAFi). Forty-eight hours later cells were quantified. (C) A375-GFP cells isolated from mice treated as shown in Figure 1C were cultured in the presence of vemurafenib (BRAFi) or selumetinib (MEKi) alone or in combination with nelfinavir for 3 weeks before quantification. Data show box plots indicating the upper/lower quartile and the median with whiskers from min to max values. (D) GFP-expressing A375 cells (false colored in red) were injected into zebrafish larvae; the larvae were treated with DMSO, PD184352 (MEKi), or nelfinavir alone or in combination. Three days after drug addiction the xenografts were imaged (scale bars, 100 μm) and the volume was quantified using Volocity software. Data show scatter dot plots, indicating the mean ± SD ^∗^p < 0.05. (E) MITF immunofluorescence analysis of A375 tumors from mice treated with vehicle, nelfinavir (25 mg/kg qd) or PLX4720 (BRAFi, 25 mg/kg qd) alone or in combination for 21 days; scale bars, 200 μm. (F) qRT-PCR for PAX3 and MITF expression in the individual tumors; mean expression ± SEM relative to vehicle control, ^∗∗^p < 0.01; ^∗∗∗^p < 0.001. (G) Mean tumor volumes ± SEM (n = 8) and a phospho-ERK IHC for a vehicle tumor and PLX4720 (BRAFi; 25 mg/kg)-treated tumor; scale bars, 200 μm. ^∗∗^p < 0.01; ^∗∗∗^p < 0.001. See also Figure S6.

Because nelfinavir efficiently suppresses PAX3 and MITF expression, we wanted to assess its function in MAPK-pathway-targeting therapy. MITF is a crucial regulator of G_1_/S transition, and accordingly PAX3 and MITF depletion as well as nelfinavir treatment resulted in a G_1_ arrest (Figures S6A and S6B). However, MAPKi combination treatment induced cell death and reduced cell numbers in a synergistic manner in *BRAF* mutant melanoma cell lines, but not in the *BRAF* mutant/MITF-negative colon cancer cell line RKO (Figures 6A and 6B). Furthermore, the GI_50_ for combination treatments increased with enhanced PAX3 and MITF expression (Figure S6C). The presence of nelfinavir during a 3-week treatment of drug-tolerant A375-GFP cells #026 and #549 (isolated from BRAF inhibitor-treated mice, see Figure 1C) with MAPKis overcame the development of resistant clones (Figure 6C). In a short-term zebrafish xenograft assay, combination treatment resulted in tumor volume reduction (Figure 6D), demonstrating that nelfinavir can sensitize to the cytotoxic effects of the inhibitor in vivo. However, the time frame of this experiment does not allow assessing the tolerance phase during which we have observed PAX3 and MITF upregulation. We therefore treated mice bearing A375 xenografts with nelfinavir for a period of 3 weeks. During this time, as seen previously, BRAF inhibition induced a profound upregulation of both PAX3 and MITF expression (Figures 6E and 6F). While nelfinavir treatment alone produced a slight reduction in PAX3 as well as MITF expression, its combination with a BRAF inhibitor completely abolished the PAX3 and MITF upregulation (Figures 6D and 6E). This was correlated with MITF target gene expression (Figure S6D) and tumor growth, where the BRAF inhibitor/nelfinavir combination led to an over 80% reduction in tumor volume (Figure 6G).

### Nelfinavir Sensitizes *NRAS* Mutant Melanoma to MEK Inhibition

**Figure 7:**
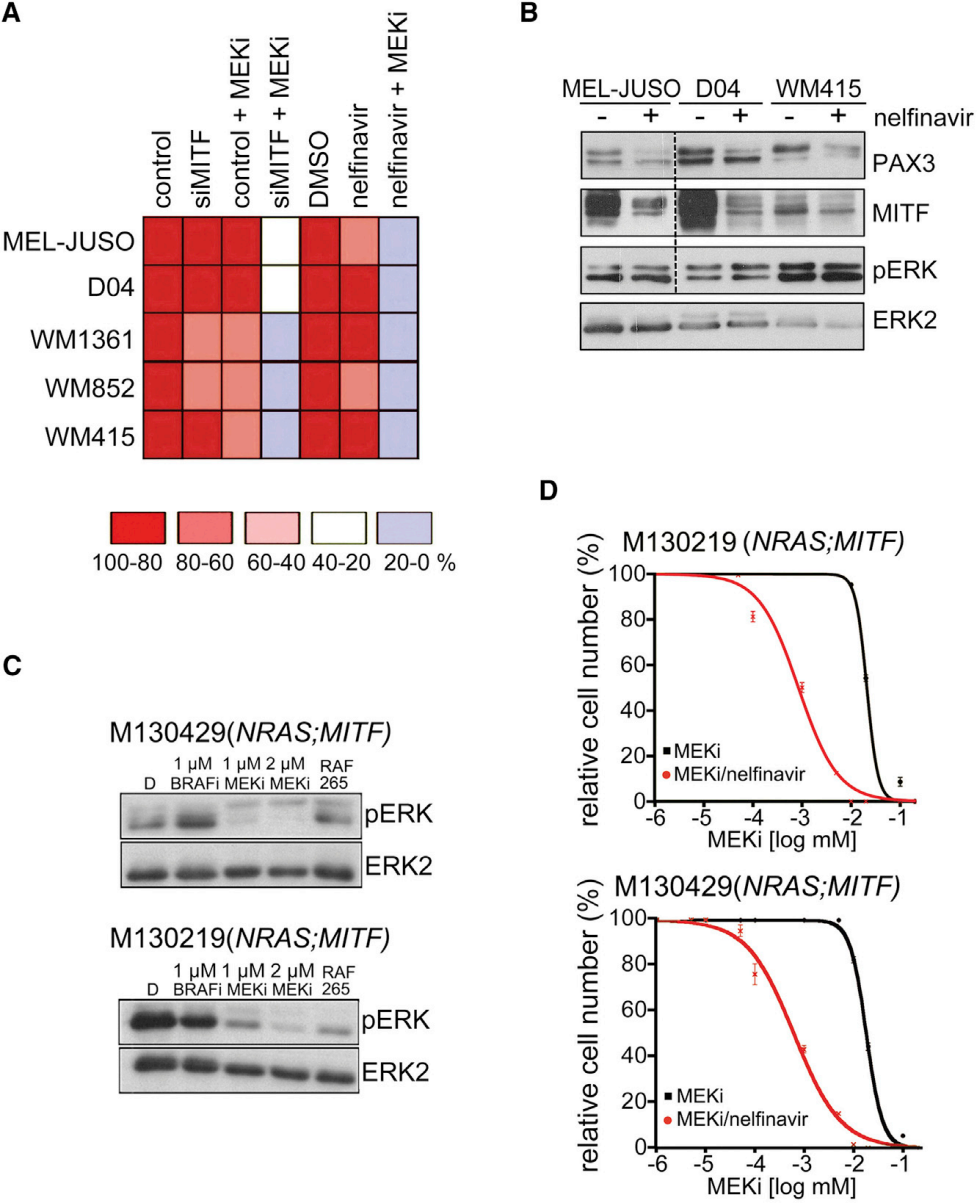
Nelfinavir Sensitizes *NRAS* Mutant Melanoma to MEK Inhibition (A) Summary of survival of indicated cell lines treated with MITF siRNA nelfinavir (10 μM for 24 hr) or DMSO, followed by 48-hr incubation with selumetinib (MEKi). Cells were quantified using crystal violet. (B) Western blot of *NRAS* mutant melanoma cells treated with DMSO or 10 μM nelfinavir for 24 hr for PAX3, MITF pERK, and ERK2. MEL-JUSO samples for PAX3, MITF, and pERK detection corresponding to the loading control ERK2 were run on a separate gel. (C) M130219 and M130429 cells treated with vemurafenib (BRAFi) or selumetinib (MEKi) or RAF265 for 48 hr were analyzed by western blotting for pERK and ERK2. (D) Dose-response curves (mean ± SEM) for M130219 and M130429 cells treated with MEKi (selumetinib) in the presence or absence of 5 μM nelfinavir for 72 hr. See also Table S2.

Because MITF is crucial for the survival of the melanocyte lineage, we argued that it would also be relevant for *NRAS* mutant melanoma cell survival. Indeed, the depletion of MITF from MITF-expressing *NRAS* mutant melanoma cells significantly sensitized these cells to MEK inhibition (Figure 7A). Nelfinavir also sensitized *NRAS* mutant melanoma cells to MEK inhibition and reduced PAX3 and MITF expression, whereas no sensitization was seen in the *KRAS* mutant colon cancer cell line HCT116 (Figures 6B, 7A, and 7B). We next tested two short-term cultures from a patient with *NRAS*^Q61K^ mutant melanoma progressed on MEK inhibitor treatment. This patient also carried an *MITF*^E318K^ germline mutation, which is linked to increased melanoma susceptibility (Table S2). Both cultures still responded to MEK inhibitor with reduced ERK phosphorylation (Figure 7C), suggesting that the resistance had developed by acquiring additional survival advantages. Nelfinavir treatment profoundly sensitized the growth of these cultures to MEK inhibition (Figure 7D).

### Nelfinavir Overcomes Mutant *NRAS*-Mediated Acquired Resistance

**Figure 8:**
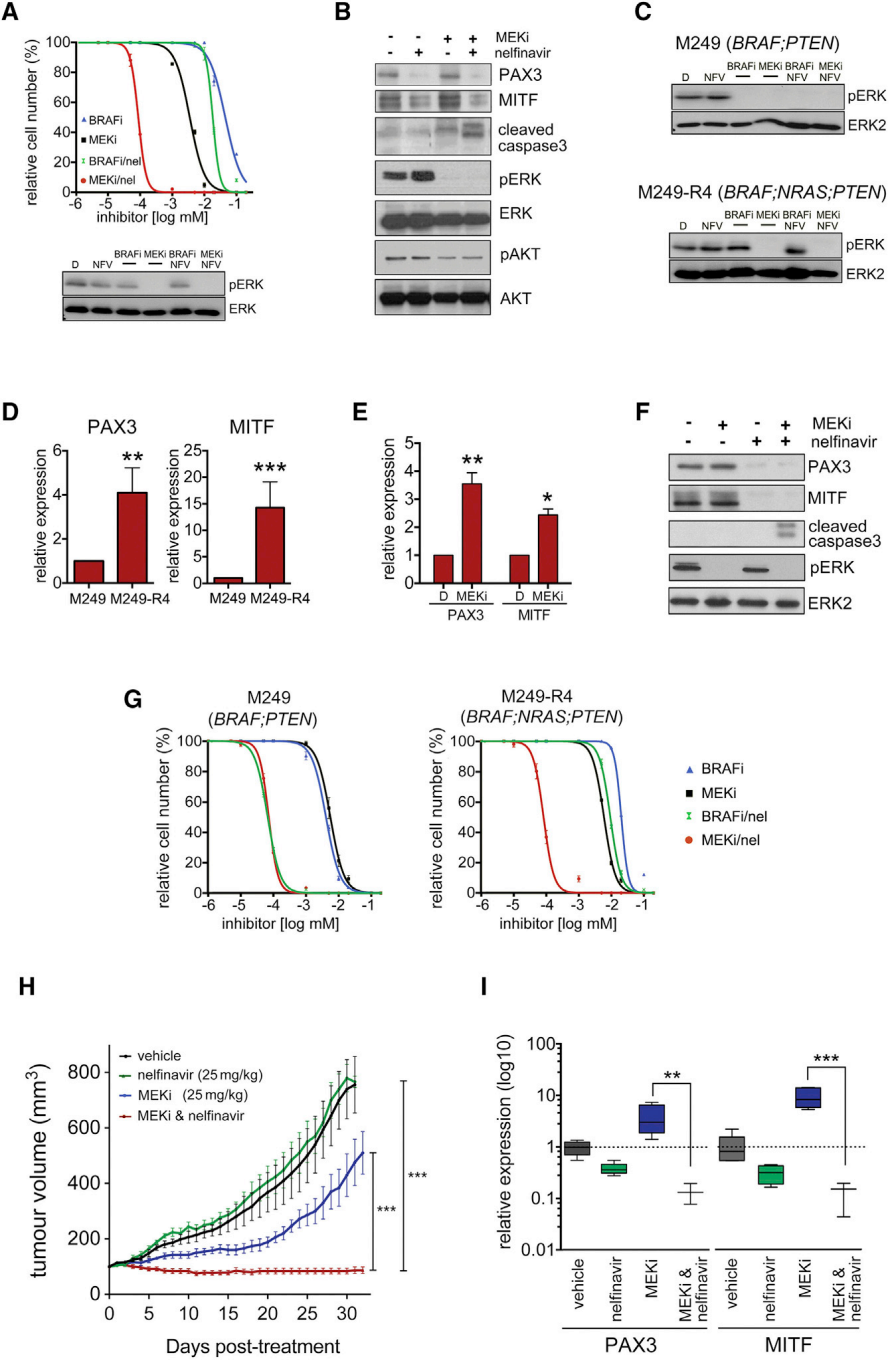
Nelfinavir Overcomes *NRAS*-Driven Acquired Resistance (A) Dose-response curve (mean ± SEM) for M121224 cells treated for 48 hr with vemurafenib (BRAFi) or selumetinib (MEKi) in the presence or absence of 5 μM nelfinavir for 72 hr. DMSO-treated cells were set 100%. Western blot for pERK and ERK2 of M121224 cells treated as indicated above. (B) Western blot of M11224 cells incubated with nelfinavir (10 μM) alone or 48 hr in combination with selumetinib (MEKi) for the indicated proteins. (C) M249 and M249-R4 cells treated with vemurafenib (BRAFi) or selumetinib (MEKi) for 48 hr alone or in the presence of nelfinavir (10 μM) were analyzed by western blotting for pERK and ERK2 (D) qRT-PCR analysis for PAX3 and MITF expression (mean ± SEM) in M249 and M249-R4 cells. (E) qRT-PCR analysis for PAX3 and MITF expression (mean ± SEM) in M249-R4 cells treated with DMSO or trametinib (MEKi) for 24 hr. (F) Western blot for PAX3, MITF, cleaved caspase3, pERK, and ERK2 of M249-R4 cells treated with selumetinib (MEKi) or nelfinavir (10 μM) for 48 hr alone or in combination. (G) Dose-response curves (mean ± SEM) for M249 and M249-R4 cells treated with vemurafenib (BRAFi) or selumetinib (MEKi) in the presence or absence of 5 μM nelfinavir for 72 hr. DMSO-treated cells were set 100%. (H) Nude mice bearing tumors from M249-R4 cells were treated with vehicle, nelfinavir (25 mg/kg qd) or selumetinib (25 mg/kg qd) alone or in combination for 33 consecutive days. The results show mean tumor volumes ± SEM (n = 6). (I) qRT-PCR for PAX3 and MITF expression in the individual tumors. Data show box plots indicating the upper/lower quartile and the median with whiskers from min to max values. For all graphs: ^∗^p < 0.05; ^∗∗^p < 0.01; ^∗∗∗^p < 0.001. See also Figure S7.

Mutated *NRAS* is found in ∼18% of melanomas with acquired resistance (Shi et al., 2014, Van Allen et al., 2014). Confirming previous observations (Nazarian et al., 2010), we found that in a short-term culture derived from a BRAF-inhibitor-treated patient, who progressed with a *NRAS*^Q61K^ mutation (Table S2), a BRAF inhibitor was not efficient in inhibiting ERK phosphorylation (Figure 8A). MEK inhibition, however, blocked ERK phosphorylation and was ∼10 times more effective in reducing cell growth than BRAF inhibition (Figure 8A). However, the presence of nelfinavir increased cell killing by ∼500-fold compared with BRAF inhibition (Figure 8A). This increased cytotoxic effect was also seen at the level of caspase3 cleavage, demonstrating that nelfinavir enhances the cytotoxic effects of MEK inhibition (Figure 8B).

We have shown that nelfinavir sensitizes to MAPKi under basal growth conditions, but also counteracts the MAPKi-induced upregulation of PAX3 and MITF, which we detect in tumors on treatment. However, some tumors progress with increased levels of MITF expression (see Figure 5G). For the *BRAF*^V600E^;*NRAS*^Q61K^ culture, we did not have a paired “before” culture and hence could not assess whether PAX3 or MITF expression was increased in the culture from the acquired resistant tumor. We therefore moved to a more controlled system and used the previously described in vitro generated resistant M249-R4 cells, which are derived from *BRAF*^V600E^;*PTEN*^−/-^ M249 cells (Nazarian et al., 2010). In *NRAS*^Q61K^-expressing M249-R4 cells, ERK activation by MEK is resistant to BRAF inhibition, but the cells still respond to MEK inhibition (Figure 8C). M249-R4 cells express higher levels of PAX3 and MITF than M249 cells (Figure 8D), but MEK inhibition still results in upregulation of PAX3 and MITF mRNA (Figure 8E), whereas nelfinavir reduces PAX3 and MITF expression (Figure 8F). While nelfinavir strongly sensitizes M249 to BRAF inhibitor, M249-R4 cells do not display a major response (Figure 8G), further confirming their resistance to BRAF inhibition. However, nelfinavir increased the sensitivity to MEK inhibition not only in M249 but also in M249-R4 cells, where the GI_50_ was shifted almost 100-fold when nelfinavir was present (Figure 8G). Most importantly however, this sensitization was also seen in vivo, where the MEK inhibitor/nelfinavir combination completely suppressed tumor growth, even when tumors started to progress on MEK inhibitor monotherapy (Figure 8H). This was reflected in PAX3, MITF, as well as MITF target gene expression (Figures 8I and S7), demonstrating that reduced MITF function is linked to repressed tumor growth.

## Discussion

The mutational heterogeneity found in tumors of patients progressed on targeted therapy implies that additional strategies to tackle reduced responsiveness to small molecule inhibitor treatment should be considered. Our data suggest that targeting a non-mutational tolerance phase preceding acquired mutational resistance can be such a strategy.

Drug-induced tolerance has been reported in cell lines from various cancer cell types after long-term in vitro treatment, and this has been linked to chromatin modifications (Sharma et al., 2010). Comparable observations were made in melanoma cell lines treated with sub-lethal concentrations of vemurafenib (Menon et al., 2015). This resulted in chromatin modifications paralleled by a distinct expression program involving the upregulation of stem cell markers and downregulation of differentiation markers like *MLANA* and *TYR*, which is in line with downregulation of MITF. A similar response might have occurred in the tumors of the two patients, where we observed a reduction in MITF expression. We do not know whether the lower frequency of this response within a 2-week time frame reflects that losing MITF expression is not due to a direct signaling response to MAPK-pathway inhibition, and might be either an indirect consequence of overall changes in the epigenetic state over time or enrichment of MITF-negative cell populations on treatment. Considerably more samples from patients on treatment will be required to validate the frequency of MITF reduction and to address these questions.

Nevertheless, in line with others (Gopal et al., 2014, Ji et al., 2015), we find reduced MITF expression in ∼50% of tumors on progression. So far it is unclear what triggers this response, because we and others find that the other 50% of acquired resistant tumors not only display MITF expression levels comparable with before treatment, a fraction of patients also relapse with tumors having greatly increased MITF (Gopal et al., 2014, Ji et al., 2015), which might be the result of *MITF* amplification (Van Allen et al., 2014). We found upregulated MITF expression in the *NRAS*^Q61K^-driven M249-R4 cells, and although we do not know whether MITF can drive acquired resistance, we show that targeting MITF in acquired resistant cells can sensitize them to MAPK inhibitors.

Predominant upregulation of *MLANA* and other MITF target genes within the first 14 days of treatment with BRAF inhibitor monotherapy has also been reported in another cohort of patients (Frederick et al., 2013). Moreover, while the expression of these melanoma differentiation antigens was back to basal level in patients on progression, in a patient who then was treated with a BRAF/MEK inhibitor combination, the MITF target genes were again upregulated in response to treatment (Frederick et al., 2013). This strongly supports the reversible non-mutational nature of these changes, and also further suggests that MITF upregulation is a fairly uniform response.

The upregulation of MITF was paralleled by the upregulation of the transcriptional regulator PAX3 (Kubic et al., 2008). In adult melanocytes as well as in development, PAX3 expression is suppressed by TGF-β signaling, and the transcriptional co-repressor SKI plays a crucial role in this suppression (Xu et al., 2000, Yang et al., 2008). We identified SKI as an MAPK-regulated suppressor of PAX3 in melanoma cells. SKI is an important regulator of melanoma growth (Chen et al., 2009), and the elevated MAPK-pathway activity found in melanomas might contribute to its increased expression. By suppressing PAX3, SKI counteracts the positive regulation of the *MITF* promoter induced by *BRAF*^V600E^ through BRN2 (Wellbrock et al., 2008). SKI thus helps to maintain the MITF homeostasis downstream of *BRAF*^V600E^ required for BRAF-driven melanoma development (Wellbrock and Arozarena, 2015).

We demonstrate that the HIV1-protease inhibitor nelfinavir targets PAX3 expression by increasing the SMAD2/4/SKI suppressor complex. Nelfinavir induces an increase in SMAD2 levels within 6 hr, but we have not yet identified the upstream regulator of this event. Nelfinavir can inhibit the proteasome (Gupta et al., 2007), which could slow down the turnover of SMAD2. However, this is unlikely to be the case in melanoma cells, where proteasome inhibition very effectively increases MITF levels (Wellbrock and Marais, 2005, Wu et al., 2000), as we observe efficient downregulation of MITF. While in various cancer types nelfinavir targets AKT signaling, the underlying mechanisms in this context are still a matter of debate (Shim and Liu, 2014). Moreover, a previous study analyzing a panel of *BRAF*^V600E^ melanoma cells did not detect any reduction in AKT phosphorylation (Jiang et al., 2007), and we confirm these previous observations. Overall, data concerning the mode of action of nelfinavir are conflicting and also appear to be dependent on the cancer cell type.

Nelfinavir induces cell death in various cancer cell lines partly by triggering ER stress and autophagy (Gills et al., 2007). However, in melanoma cells we observed a cell-cycle arrest as initial response, which confirms previous findings of nelfinavir inducing a G_1_ arrest and suppressing CDK2 activity in melanoma cells (Jiang et al., 2007). The latter is striking, because *CDK2* itself is an important MITF target gene (Du et al., 2004). Moreover MITF controls the expression and activity of G_1_/S transition regulators such as p21, p27, and RB (Wellbrock and Arozarena, 2015), all of which have been described to be affected by nelfinavir in melanoma cells (Jiang et al., 2007).

The concept of repositioning HIV protease inhibitors such as nelfinavir for cancer therapy has become of interest ever since anti-neoplastic activities have been observed with these agents (Shim and Liu, 2014). Particularly nelfinavir has growth inhibitory activity in a variety of different cancer types in vitro and in vivo, and several clinical trials testing nelfinavir are currently ongoing (Shim and Liu, 2014). While the peak plasma concentration of nelfinavir in HIV patients is around 8 μM (Markowitz et al., 1998), using higher doses (without approaching the maximum tolerated dose) can lead to plasma concentrations around 5–15 μM (Gantt et al., 2013, Pan et al., 2012). Although we do not know the concentration we achieve in mice at 25 mg/kg/day, the levels reached in patients are in the range of the concentration we used (5–10 μM) to sensitize melanoma cells to BRAF or MEK inhibitor-induced cell death in vitro.

In summary, we show that inhibiting MITF expression by nelfinavir has a potent enhancer effect on the action of BRAF and MEK inhibitors. Moreover, even in cells that do not display elevated expression of MITF, its relevance for melanoma cell survival appears to be sufficient for inhibitor sensitization. Our data suggest that apart from increasing initial responses also in *NRAS* mutant patients, the nelfinavir/MEK inhibitor combination could restore the MAPK inhibitor response in patients relapsed with increased NRAS signaling. Thus, by targeting a cancer-type-specific master regulator that plays an important role in the initial phases of drug-induced tolerance, we identify a clinical relevant approach for melanoma therapy.

## Experimental Procedures

For more detailed information see Supplemental Experimental Procedures.

### Patient Samples and In Vivo Work

Patients with mutant *BRAF*^V600^-positive metastatic melanoma were treated with either a BRAF inhibitor, or a combination of BRAF and MEK inhibitors (for patient characteristics see Table S1). All patients consented for tissue acquisition as per an institutional-review-board-approved protocol (Office for Human Research Studies, Dana-Farber/Harvard Cancer Center). Tumor biopsies were obtained before treatment (day 0), at 10–14 days on treatment, and/or at time of progression if applicable. All animal procedures involving animals were ethically approved by University of Manchester Animal Welfare and Ethical Review Bodies (AWERB) and carried out under license in accordance with the UK Home Office Animals (Scientific Procedures) Act (1986) and guidelines of the Committee of the National Cancer Research Institute.

### RNA Isolation and qPCR Analysis

RNA from cell lines or frozen tumor tissue was isolated with TRIZOL and selected genes were amplified by qRT-PCR using either SYBR green (Qiagen) or TaqMan probes.

### Cell Culture Treatments and Drug Dose-Response Analysis

All melanoma cell lines were grown in Dulbecco’s modified Eagle’s medium/10% fetal calf serum (PAA). Cell numbers were measured as the optical density at 595 nm (OD_595_) of solubilized crystal violet from formalin-fixed cells. For all in vitro experiments vemurafenib was used as BRAF inhibitor. Different MEK inhibitors (PD184352, selumetinib, and trametinib) were used and are specified in the figure legends. For dose-response curves, cells were plated in 96-well plates and treated with serial dilutions of the indicated drugs. The GI_50_ was calculated using GraphPad Prism version 6.00.

### Cell Lysis and Immunoblotting

Cells were lysed in SDS sample buffer and analyzed by standard western blotting protocols. Primary antibodies were as follows: MITF (C5), Neomarkers, Lab Vision; phospho-ERK (MAPK-YT), Sigma; ERK2 (C-14), PAX3 (N-19), BRAF (F-7), and SKI (H-329), Santa Cruz Biotechnology; and SMAD2, pSMAD2, SMAD4, pAKT (S473), and Caspase 3, Cell Signaling Technology.

### Statistical Analysis

Data represent the results for assays performed in triplicate, with error bars to represent SD or SEM. Statistics used were as follows: predominately Student’s t test and one-way ANOVA with Tukeys's post hoc test performed using GraphPad Prism version 6.00 for Mac OS, GraphPad Software. Pearson correlation was used to analyze associated gene expression and Wilcoxon-Mann-Whitney test to analyze tumor growth. Throughout the text: ^∗^p < 0.05; ^∗∗^p < 0.01; ^∗∗∗^p < 0.001.

## Author Contributions

Conceptualization, M.P.S. and C.W.; Methodology, M.P.S., H.B., E.R., I.A; Validation, M.P.S., H.B., J.F.; Formal Analysis, Z.M., M.P.S., H.B., E.R., I.A., J.L., M.R.G., C.W.; Investigation, M.P.S., H.B., E.R., J.F., I.A, J.L., M.R.G; Writing – Original Draft, C.W.; Writing – Review & Editing, M.P.S. and C.W.; Supervision, C.W.; Funding Acquisition, C.W.; Resources, R.M., M.P.L., R.D., J.A.W., D.T.F., Z.A.C., K.T.F.

## Supplemental Information

Document S1. Supplemental Experimental Procedures, Figures S1–S7, and Tables S1 and S2

Document S2. Article plus Supplemental Information

## References

[bib1] Abel E.V., Basile K.J., Kugel C.H., Witkiewicz A.K., Le K., Amaravadi R.K., Karakousis G.C., Xu X., Xu W., Schuchter L.M. (2013). Melanoma adapts to RAF/MEK inhibitors through FOXD3-mediated upregulation of ERBB3. J. Clin. Invest..

[bib2] Burrell R.A., McGranahan N., Bartek J., Swanton C. (2013). The causes and consequences of genetic heterogeneity in cancer evolution. Nature.

[bib3] Cancer Genome Atlas Network (2015). Genomic classification of cutaneous melanoma. Cell.

[bib4] Chen D., Lin Q., Box N., Roop D., Ishii S., Matsuzaki K., Fan T., Hornyak T.J., Reed J.A., Stavnezer E. (2009). SKI knockdown inhibits human melanoma tumor growth in vivo. Pigment Cell Melanoma Res..

[bib5] Chow W.A., Jiang C., Guan M. (2009). Anti-HIV drugs for cancer therapeutics: back to the future?. Lancet Oncol..

[bib6] da Rocha Dias S., Friedlos F., Light Y., Springer C., Workman P., Marais R. (2005). Activated B-RAF is an Hsp90 client protein that is targeted by the anticancer drug 17-allylamino-17-demethoxygeldanamycin. Cancer Res..

[bib7] Du J., Widlund H.R., Horstmann M.A., Ramaswamy S., Ross K., Huber W.E., Nishimura E.K., Golub T.R., Fisher D.E. (2004). Critical role of CDK2 for melanoma growth linked to its melanocyte-specific transcriptional regulation by MITF. Cancer Cell.

[bib8] Frederick D.T., Piris A., Cogdill A.P., Cooper Z.A., Lezcano C., Ferrone C.R., Mitra D., Boni A., Newton L.P., Liu C. (2013). BRAF inhibition is associated with enhanced melanoma antigen expression and a more favorable tumor microenvironment in patients with metastatic melanoma. Clin. Cancer Res..

[bib9] Gantt S., Casper C., Ambinder R.F. (2013). Insights into the broad cellular effects of nelfinavir and the HIV protease inhibitors supporting their role in cancer treatment and prevention. Curr. Opin. Oncol..

[bib10] Gills J.J., Lopiccolo J., Tsurutani J., Shoemaker R.H., Best C.J., Abu-Asab M.S., Borojerdi J., Warfel N.A., Gardner E.R., Danish M. (2007). Nelfinavir, A lead HIV protease inhibitor, is a broad-spectrum, anticancer agent that induces endoplasmic reticulum stress, autophagy, and apoptosis in vitro and in vivo. Clin. Cancer Res..

[bib11] Girotti M.R., Pedersen M., Sanchez-Laorden B., Viros A., Turajlic S., Niculescu-Duvaz D., Zambon A., Sinclair J., Hayes A., Gore M. (2013). Inhibiting EGF receptor or SRC family kinase signaling overcomes BRAF inhibitor resistance in melanoma. Cancer Discov..

[bib12] Gopal Y.N., Deng W., Woodman S.E., Komurov K., Ram P., Smith P.D., Davies M.A. (2010). Basal and treatment-induced activation of AKT mediates resistance to cell death by AZD6244 (ARRY-142886) in Braf-mutant human cutaneous melanoma cells. Cancer Res..

[bib13] Gopal Y.N., Rizos H., Chen G., Deng W., Frederick D.T., Cooper Z.A., Scolyer R.A., Pupo G., Komurov K., Sehgal V. (2014). Inhibition of mTORC1/2 overcomes resistance to MAPK pathway inhibitors mediated by PGC1alpha and oxidative phosphorylation in melanoma. Cancer Res..

[bib14] Gupta A.K., Li B., Cerniglia G.J., Ahmed M.S., Hahn S.M., Maity A. (2007). The HIV protease inhibitor nelfinavir downregulates Akt phosphorylation by inhibiting proteasomal activity and inducing the unfolded protein response. Neoplasia.

[bib15] Haq R., Shoag J., Andreu-Perez P., Yokoyama S., Edelman H., Rowe G.C., Frederick D.T., Hurley A.D., Nellore A., Kung A.L. (2013). Oncogenic BRAF regulates oxidative metabolism via PGC1alpha and MITF. Cancer Cell.

[bib16] Haq R., Yokoyama S., Hawryluk E.B., Jonsson G.B., Frederick D.T., McHenry K., Porter D., Tran T.N., Love K.T., Langer R. (2013). BCL2A1 is a lineage-specific antiapoptotic melanoma oncogene that confers resistance to BRAF inhibition. Proc. Natl. Acad. Sci. USA.

[bib17] Ji Z., Erin Chen Y., Kumar R., Taylor M., Jenny Njauw C.N., Miao B., Frederick D.T., Wargo J.A., Flaherty K.T., Jonsson G., Tsao H. (2015). MITF modulates therapeutic resistance through EGFR signaling. J. Invest. Dermatol..

[bib18] Jiang W., Mikochik P.J., Ra J.H., Lei H., Flaherty K.T., Winkler J.D., Spitz F.R. (2007). HIV protease inhibitor nelfinavir inhibits growth of human melanoma cells by induction of cell cycle arrest. Cancer Res..

[bib19] Johannessen C.M., Johnson L.A., Piccioni F., Townes A., Frederick D.T., Donahue M.K., Narayan R., Flaherty K.T., Wargo J.A., Root D.E., Garraway L.A. (2013). A melanocyte lineage program confers resistance to MAP kinase pathway inhibition. Nature.

[bib20] Kubic J.D., Young K.P., Plummer R.S., Ludvik A.E., Lang D. (2008). Pigmentation PAX-ways: the role of Pax3 in melanogenesis, melanocyte stem cell maintenance, and disease. Pigment Cell Melanoma Res..

[bib21] Larkin J., Ascierto P.A., Dreno B., Atkinson V., Liszkay G., Maio M., Mandala M., Demidov L., Stroyakovskiy D., Thomas L. (2014). Combined vemurafenib and cobimetinib in BRAF-mutated melanoma. N. Engl. J. Med..

[bib22] Lito P., Pratilas C.A., Joseph E.W., Tadi M., Halilovic E., Zubrowski M., Huang A., Wong W.L., Callahan M.K., Merghoub T. (2012). Relief of profound feedback inhibition of mitogenic signaling by RAF inhibitors attenuates their activity in BRAFV600E melanomas. Cancer Cell.

[bib23] Long G.V., Fung C., Menzies A.M., Pupo G.M., Carlino M.S., Hyman J., Shahheydari H., Tembe V., Thompson J.F., Saw R.P. (2014). Increased MAPK reactivation in early resistance to dabrafenib/trametinib combination therapy of BRAF-mutant metastatic melanoma. Nat. Commun..

[bib24] Long G.V., Stroyakovskiy D., Gogas H., Levchenko E., de Braud F., Larkin J., Garbe C., Jouary T., Hauschild A., Grob J.J. (2015). Dabrafenib and trametinib versus dabrafenib and placebo for Val600 BRAF-mutant melanoma: a multicentre, double-blind, phase 3 randomised controlled trial. Lancet.

[bib25] Markowitz M., Conant M., Hurley A., Schluger R., Duran M., Peterkin J., Chapman S., Patick A., Hendricks A., Yuen G.J. (1998). A preliminary evaluation of nelfinavir mesylate, an inhibitor of human immunodeficiency virus (HIV)-1 protease, to treat HIV infection. J. Infect Dis..

[bib26] Menon D.R., Das S., Krepler C., Vultur A., Rinner B., Schauer S., Kashofer K., Wagner K., Zhang G., Rad E.B. (2015). A stress-induced early innate response causes multidrug tolerance in melanoma. Oncogene.

[bib27] Muller J., Krijgsman O., Tsoi J., Robert L., Hugo W., Song C., Kong X., Possik P.A., Cornelissen-Steijger P.D., Foppen M.H. (2014). Low MITF/AXL ratio predicts early resistance to multiple targeted drugs in melanoma. Nat. Commun..

[bib28] Nazarian R., Shi H., Wang Q., Kong X., Koya R.C., Lee H., Chen Z., Lee M.K., Attar N., Sazegar H. (2010). Melanomas acquire resistance to B-RAF(V600E) inhibition by RTK or N-RAS upregulation. Nature.

[bib29] Obenauf A.C., Zou Y., Ji A.L., Vanharanta S., Shu W., Shi H., Kong X., Bosenberg M.C., Wiesner T., Rosen N. (2015). Therapy-induced tumour secretomes promote resistance and tumour progression. Nature.

[bib30] Pan J., Mott M., Xi B., Hepner E., Guan M., Fousek K., Magnusson R., Tinsley R., Valdes F., Frankel P. (2012). Phase I study of nelfinavir in liposarcoma. Cancer Chemother. Pharmacol..

[bib31] Rizos H., Menzies A.M., Pupo G.M., Carlino M.S., Fung C., Hyman J., Haydu L.E., Mijatov B., Becker T.M., Boyd S.C. (2014). BRAF inhibitor resistance mechanisms in metastatic melanoma: spectrum and clinical impact. Clin. Cancer Res..

[bib32] Salama A.K., Flaherty K.T. (2013). BRAF in melanoma: current strategies and future directions. Clin. Cancer Res..

[bib33] Sharma S.V., Lee D.Y., Li B., Quinlan M.P., Takahashi F., Maheswaran S., McDermott U., Azizian N., Zou L., Fischbach M.A. (2010). A chromatin-mediated reversible drug-tolerant state in cancer cell subpopulations. Cell.

[bib34] Shi H., Hugo W., Kong X., Hong A., Koya R.C., Moriceau G., Chodon T., Guo R., Johnson D.B., Dahlman K.B. (2014). Acquired resistance and clonal evolution in melanoma during BRAF inhibitor therapy. Cancer Discov..

[bib35] Shim J.S., Liu J.O. (2014). Recent advances in drug repositioning for the discovery of new anticancer drugs. Int. J. Biol. Sci..

[bib36] Smith M.P., Ferguson J., Arozarena I., Hayward R., Marais R., Chapman A., Hurlstone A., Wellbrock C. (2013). Effect of SMURF2 targeting on susceptibility to MEK inhibitors in melanoma. J. Natl. Cancer Inst..

[bib37] Sun C., Wang L., Huang S., Heynen G.J., Prahallad A., Robert C., Haanen J., Blank C., Wesseling J., Willems S.M. (2014). Reversible and adaptive resistance to BRAF(V600E) inhibition in melanoma. Nature.

[bib38] Van Allen E.M., Wagle N., Sucker A., Treacy D.J., Johannessen C.M., Goetz E.M., Place C.S., Taylor-Weiner A., Whittaker S., Kryukov G.V. (2014). The genetic landscape of clinical resistance to RAF inhibition in metastatic melanoma. Cancer Discov..

[bib39] von Kriegsheim A., Baiocchi D., Birtwistle M., Sumpton D., Bienvenut W., Morrice N., Yamada K., Lamond A., Kalna G., Orton R. (2009). Cell fate decisions are specified by the dynamic ERK interactome. Nat. Cell Biol..

[bib40] Wellbrock C., Arozarena I. (2015). Microphthalmia-associated transcription factor in melanoma development and MAP-kinase pathway targeted therapy. Pigment Cell Melanoma Res..

[bib41] Wellbrock C., Marais R. (2005). Elevated expression of MITF counteracts B-RAF-stimulated melanocyte and melanoma cell proliferation. J. Cell Biol..

[bib42] Wellbrock C., Rana S., Paterson H., Pickersgill H., Brummelkamp T., Marais R. (2008). Oncogenic BRAF regulates melanoma proliferation through the lineage specific factor MITF. PLoS One.

[bib43] Wu M., Hemesath T.J., Takemoto C.M., Horstmann M.A., Wells A.G., Price E.R., Fisher D.Z., Fisher D.E. (2000). c-Kit triggers dual phosphorylations, which couple activation and degradation of the essential melanocyte factor Mi. Genes Dev..

[bib44] Xu W., Angelis K., Danielpour D., Haddad M.M., Bischof O., Campisi J., Stavnezer E., Medrano E.E. (2000). Ski acts as a co-repressor with Smad2 and Smad3 to regulate the response to type beta transforming growth factor. Proc. Natl. Acad. Sci. USA.

[bib45] Yang G., Li Y., Nishimura E.K., Xin H., Zhou A., Guo Y., Dong L., Denning M.F., Nickoloff B.J., Cui R. (2008). Inhibition of PAX3 by TGF-beta modulates melanocyte viability. Mol. Cell.

