# Supplementary material for: Inhibiting Drivers of Non-mutational Drug Tolerance Is a Salvage Strategy for Targeted Melanoma Therapy
Source: Cancer Cell. 2016 Mar 14;29(3):270–84. doi: 10.1016/j.ccell.2016.02.003 (PMC4796027; doi:10.1016/j.ccell.2016.02.003)
Supplement: Document S1. Supplemental Experimental Procedures, Figures S1–S7, and Tables S1 and S2 [file mmc1.pdf]

Cancer Cell, Volume *29*

Supplemental Information

Inhibiting Drivers of Non-mutational Drug Tolerance Is a Salvage Strategy for Targeted Melanoma Therapy

Michael P. Smith, Holly Brunton, Emily J. Rowling, Jennifer Ferguson, Imanol Arozarena, Zsofia Miskolczi, Jessica L. Lee, Maria R. Girotti, Richard Marais, Mitchell P. Levesque, Reinhard Dummer, Dennie T. Frederick, Keith T. Flaherty, Zachary A. Cooper, Jennifer A. Wargo, and Claudia Wellbrock

Supplemental Data

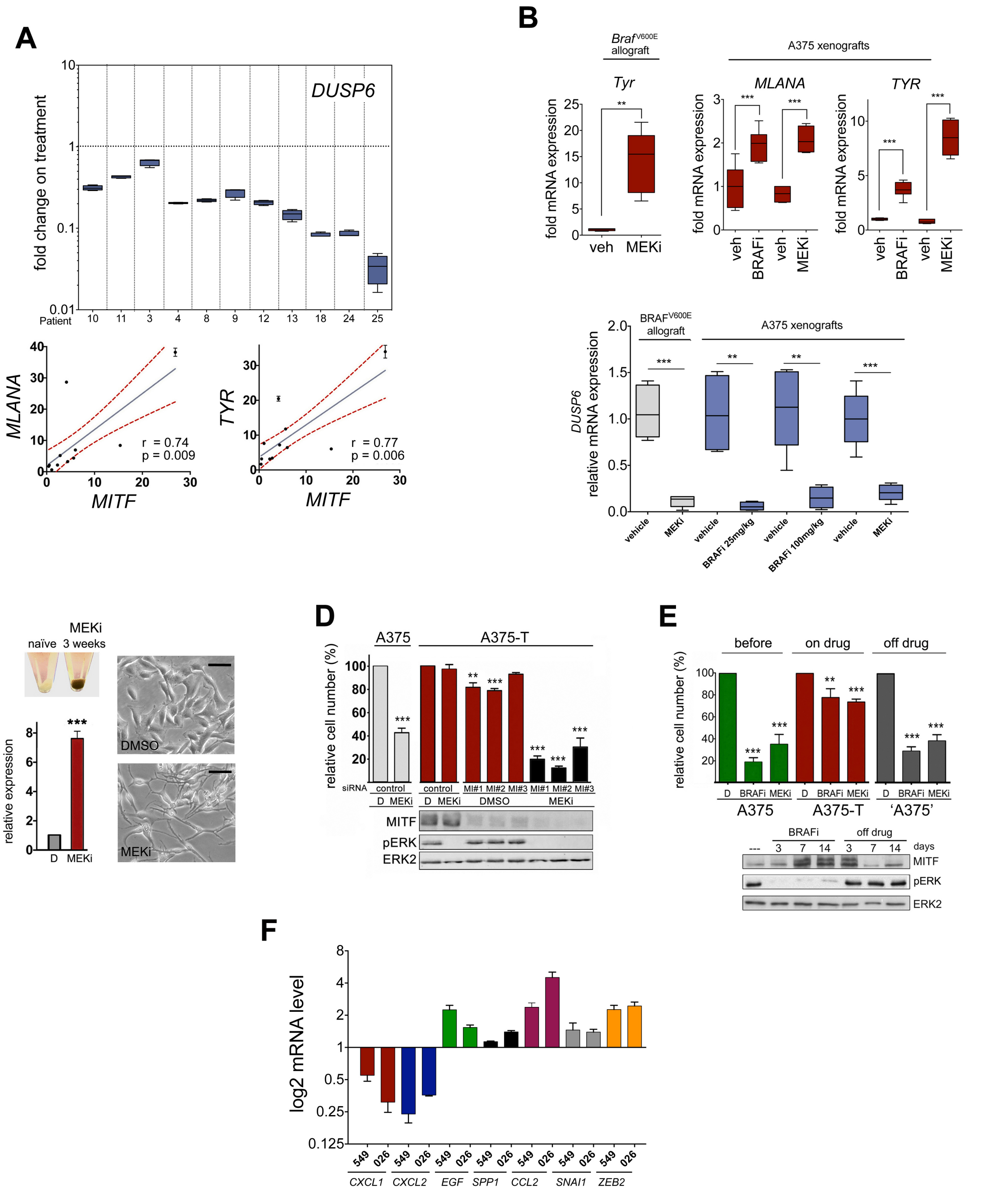

Figure S1 (related to Figure 1): MAPKi treatment induces MITF mediated gene expression and drug tolerance

(A) Correlation of *MITF* expression with *TYR* and *MLANA* expression assessed by real-time qPCR analysis in patients undergoing treatment with vemurafenib or dabrafenib/trametinib combination. *DUSP6* expression was analyzed to assess MAPK-pathway inhibition. (B) Real-time qPCR analysis of A375 xenografts and *Braf*^{V600E} allografts for *MLANA* and *TYR* and Tyr expression, respectively. *DUSP6* expression was analyzed to assess MAPK-

pathway inhibition. *Braf*^{V600E} murine melanoma allografts were from mice treated with vehicle or 25 mg/kg/qd PD184352 (MEKi) (each group: n = 5), and A375 xenografts were from mice treated with vehicle, 10 mg/kg/qd selumetinib (MEKi) or 25 mg/kg/qd PLX4720 (BRAFi) (each group: n = 3). (C) Naïve A375 cells and A375 cells treated with PD184352 (MEKi) for 3 weeks were analyzed for MITF expression by real-time qPCR. Untreated and treated cells were photographed as cell-pellets and bright-field images. Scale bars: 50 µm. (D) A375-T cells were transfected with control or MITF specific siRNAs and either left in DMSO or cultured in the presence of selumetinib (MEKi) for 72 hr, and quantified. Naïve A375 cells were used as control. Relative cell numbers and Western blots are shown. (E) A375, A375-T or A375-T cells that were cultured without drug for at least 14 days ('A375') were treated with vemurafenib (BRAFi) or selumetinib (MEKi) for 72 hr before relative cell number was assessed. A375 cells were treated with vemurafenib (BRAFi) for 14 days, followed by drug removal. MITF and phospho-ERK expression was analyzed by Western blotting at the indicated times. (F) Real-time qPCR analysis of A375-GFP cells isolated from mice treated with vemurafenib (BRAFi; #549, #026) for the indicated genes. Shown is the relative expression compared to cells isolated from vehicle treated mice. All box plots indicate the upper/lower quartile and the median with whiskers from min to max values. All other graphs show mean values ±SEM. **$p < 0.01$; ***$p < 0.001$.

Table S1 (related to Figure 1): Patient Characteristics

Patient	Mutation	Treatment	Response (maximal response in %)	Time to Progression (months)
3	BRAF	vemurafenib	SD (-10%)	10
4	BRAF	vemurafenib	PR (-56%)	3.5
8	BRAF	dabrafenib & trametinib	PR (-30%)	5
9	BRAF	dabrafenib & trametinib	PR (-45%)	7
10	BRAF	dabrafenib & trametinib	SD (-13%)	3
11	BRAF	dabrafenib & trametinib	PR (-80%)	10
12	BRAF	dabrafenib & trametinib	PR (-88.9%)	12, stopped at 20 months
13	BRAF	dabrafenib & trametinib	PR (-57.9%)	9, stroke
18	BRAF	dabrafenib & trametinib	SD (-16.5%)	6
24	BRAF	vemurafenib	PR (-53%)	2
25	BRAF	dabrafenib & trametinib	PR (-64%)	3

PR = partial response
SD = stable disease

A

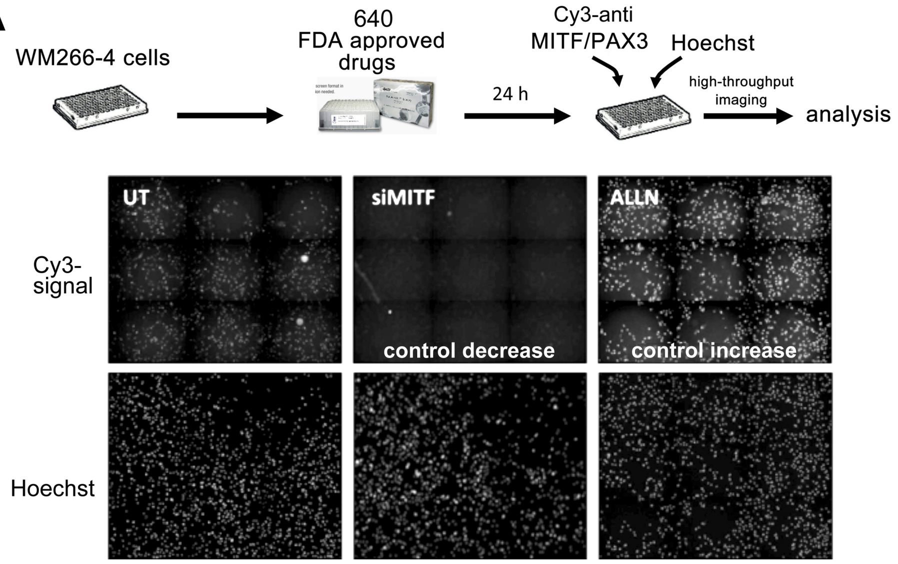

B

PAX3 z-score	MITF z-score	drug name
-10.8	-6.9	**Atazanavir** - HIV protease inhibitor
-10.5	-11.9	**Nelfinavir mesylate** - HIV protease inhibitor
-13.9	-8.1	**Spironolactone** - aldosterone R antagonist
-11.5	-9.1	**Telmisartan** - angiotensin II R antagonist
-12.5	-5.3	**Terazosin** - adrenergic antagonist
-11.5	-5.3	**Mifepristone** - steroidal (anti-glucocorticoid)
-11.7	-7.1	**Fulvestrant** - estrogen R antagonist
-13.75	-13.75	**siRNA**

Figure S2 (related to Figure 2): An FDA approved dug screen identifies nelfinavir

(A) Schematic representation of the screen protocol. WM266-4 cells were plated into 96-well plates, treated with 10μM of drug from the Enzo Life Science FDA approved library for 24 hr, then fixed stained for MITF or PAX3 using Cy3-labelled secondary antibodies, Hoechst 33342 was used to stain nuclei and define the region of interest (ROI). Cy3 signal intensity (representing MITF or PAX3 expression) per ROI was quantified using a BD pathway high-throughput microscope. Examples for the decrease control (MITF RNAi) and increase control (ALLN) are shown. The Z-factor for the PAX3 screen was 0.69 and for the MITF screen 0.88. (B) Summary of the 7 hits that targeted both PAX3 and MITF according to the set selection criteria.

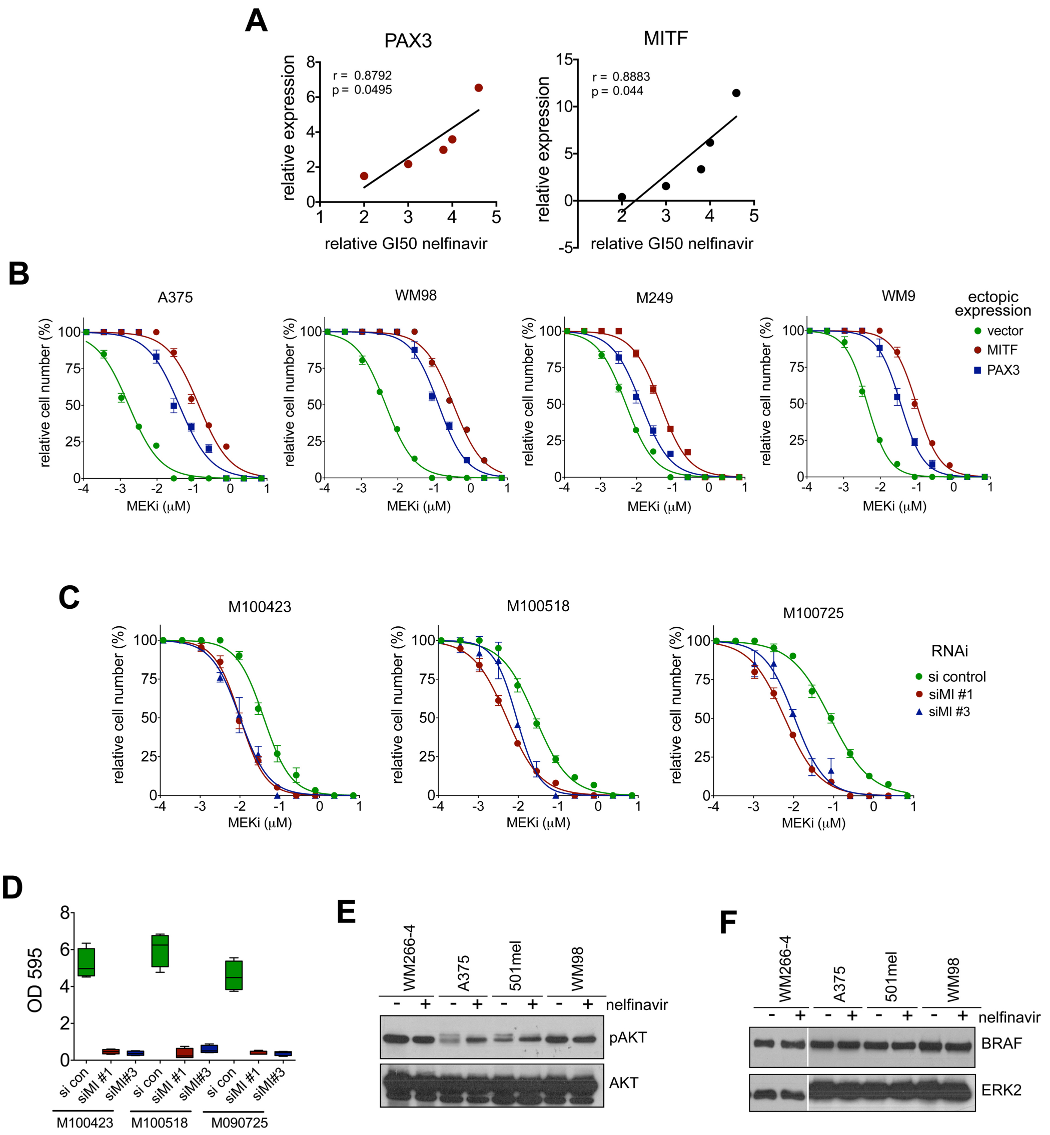

Figure S3 (related to Figure 3): MITF and PAX3 are involved in the action of nelfinavir

(A) Pearson correlation analysis for PAX3 and MITF expression and the GI_{50} values for nelfinavir in a melanoma cell line panel. (B) Dose response curves (mean ±SEM) for selumetinib (MEKi) in the presence of nelfinavir using the indicated cell lines. Cell lines had been transfected with either an empty vector or a PAX3 or MITF expressing vector. (C) Dose response curves (mean ±SEM) for selumetinib (MEKi) in the presence of nelfinavir using the indicated short-term cultures. The cells had been transfected with either a control or MITF specific siRNAs. (D) Colony survival assay for selumetinib (MEKi) treatment using the indicated short-term cultures transfected with either a control or MITF specific siRNAs. Data show box plots indicating the upper/lower quartile and the median with whiskers from min to max values. (E, F) Western blots for pAKT (E) and BRAF (F), respectively. AKT and ERK2 served as loading control.

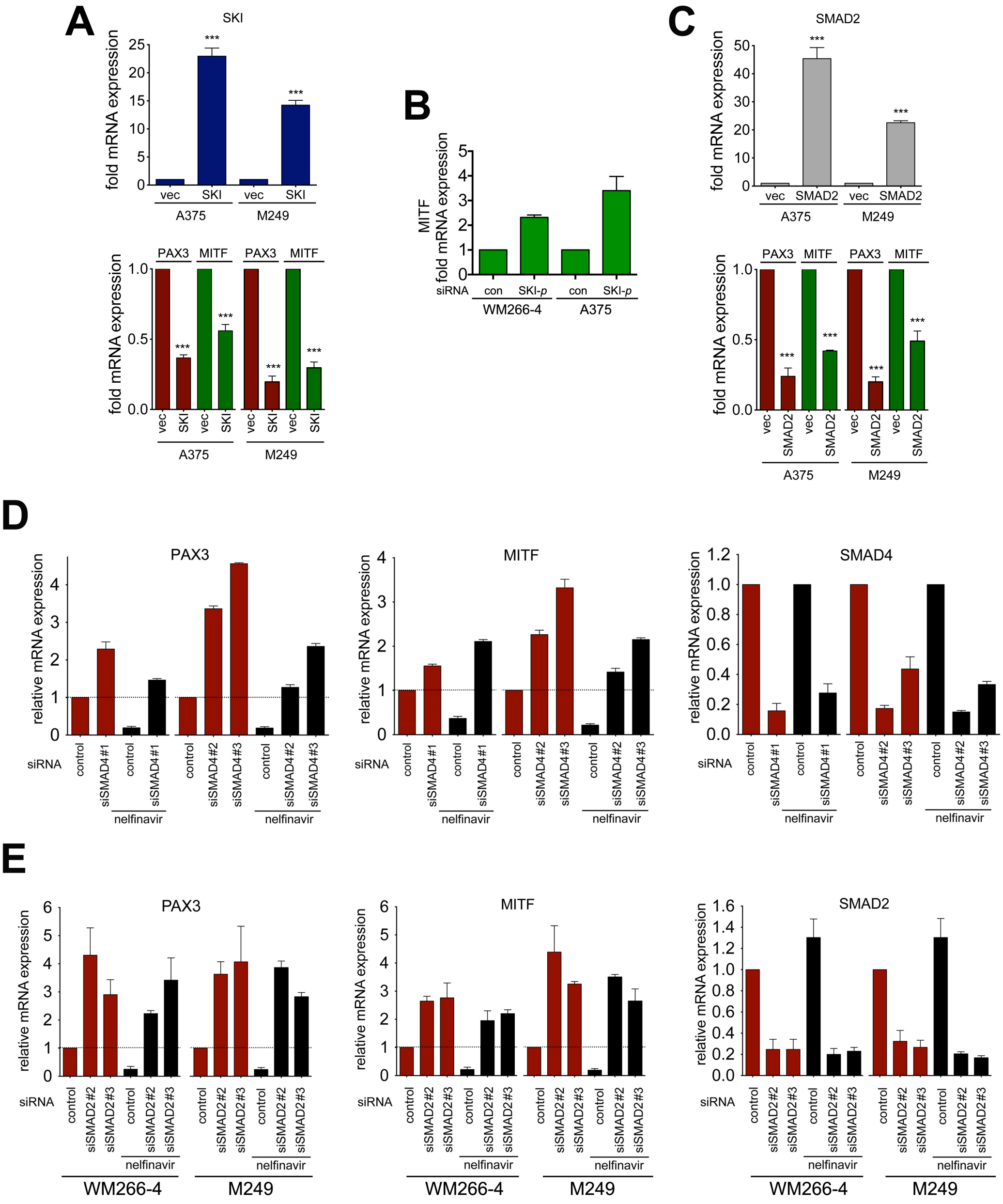

Figure S4 (related to Figure 4): SKI, SMAD2/SMAD4 regulate PAX3 expression

(A) Real-time qPCR analysis of SKI, PAX3 and MITF expression in cells transfected with either an empty vector or a SKI expression plasmid. (B) Real-time qPCR analysis of MITF expression in cells transfected with either control or a pool of 4 SKI specific siRNAs (SKI-*p*). (C) Real-time qPCR analysis of SMAD2, PAX3 and MITF expression in cells transfected with either an empty vector or a SMAD2 expression plasmid. (D) Cells were transfected with either control or three different SMAD4 specific siRNAs and either left untreated or treated with Nelfinavir. Cells were analyzed for PAX3, MITF and SMAD4 expression by real-time qPCR. (E) Cells were transfected with a control or two different SMAD2 specific siRNAs and either left untreated or treated with Nelfinavir. Cells were analyzed for PAX3, MITF and SMAD2 expression by real-time qPCR. All data indicate mean values ±SEM. ***$p < 0.001$.

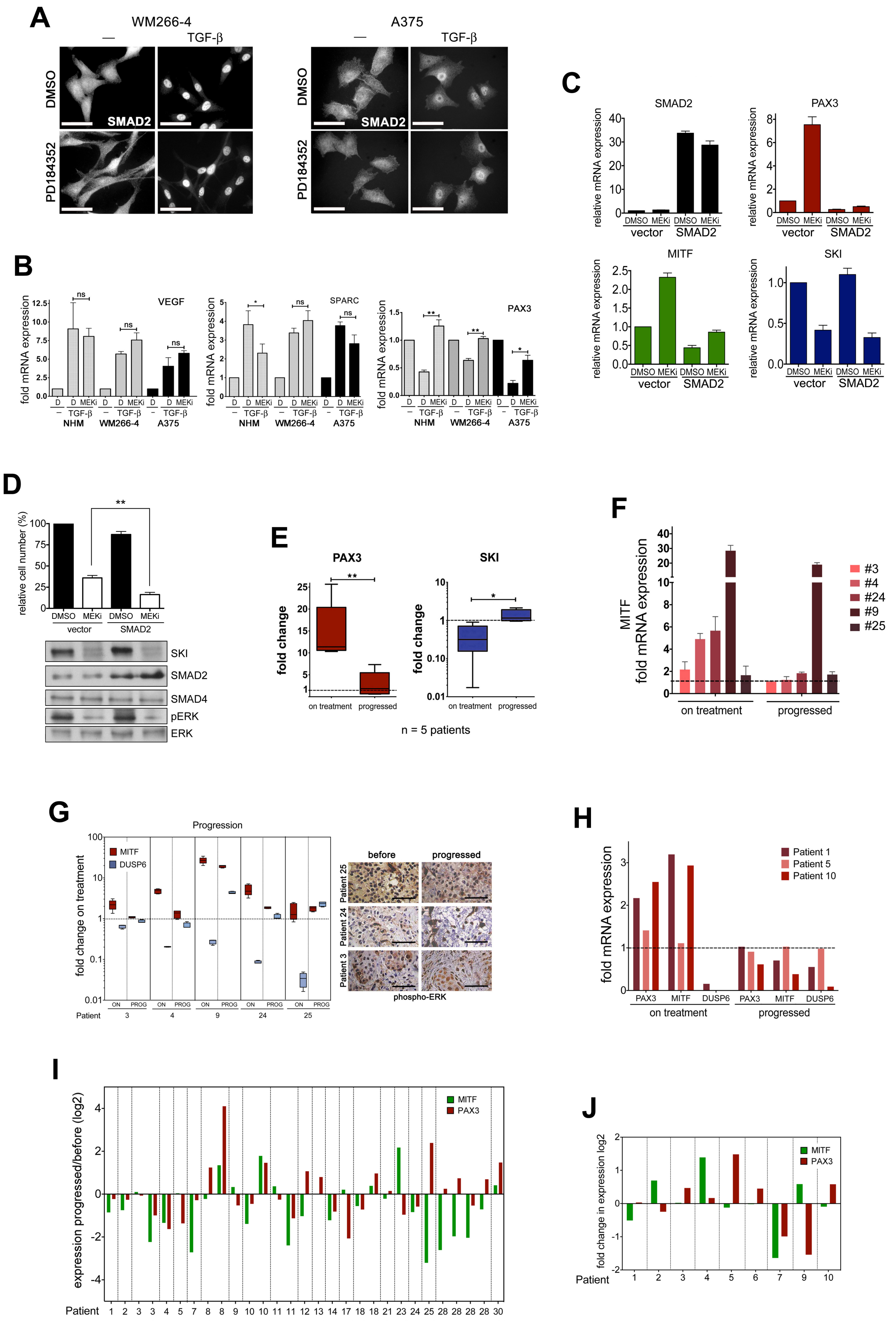

A
WM266-4
A375
TGF-β
DMSO
PD184352
SMAD2
B
VEGF
SPARC
PAX3
fold mRNA expression
NHM
WM266-4
A375
C
SMAD2
PAX3
MITF
SKI
relative mRNA expression
DMSO MEKi DMSO MEKi
vector SMAD2
D
relative cell number (%)
SKI
SMAD2
SMAD4
pERK
ERK
E
fold change
on treatment
progressed
n = 5 patients
F
MITF fold mRNA expression
#3
#4
#24
#9
#25
G
Progression
fold change on treatment
MITF
DUSP6
before
progressed
phospho-ERK
H
fold mRNA expression
Patient 1
Patient 5
Patient 10
I
expression progressed/before (log2)
J
fold change in expression log2
Patient

Figure S5 (related to Figure 5): SKI is involved in PAX3 suppression and its expression is restored in progressed tumors

(A) Immunofluorescence analysis for SMAD2 (Cy3-labbeled secondary Ab) in A375 and WM266-4 cells treated for 30 min with 5ng/ml TGF-β. Before TGF-β stimulation cells had been treated for 24 hr with DMSO or PD184352 (MEKi). Nuclei were stained with Hoechst 33342 (not shown). Scale bars: 50 µm. (B) Real-time qPCR analysis of VEGF, SPARC and PAX3 expression in melanocytes (NHM), and WM266-4 and A375 melanoma cells untreated or stimulated for 24 hr with 5ng/ml TGF-β in the presence of DMSO or PD184352 (MEKi). Data indicate the mean values ±SEM. (C) Real-time qPCR analysis of SMAD2, PAX3, MITF and SKI expression in cells transfected with either an empty vector or a SMAD2 expression plasmid. Cells were treated for 24 hr with DMSO or selumetinib (MEKi) before analysis. Data indicate the mean values ±SEM. (D) A375 cells were transfected with either an empty vector or a SMAD2 expression plasmid. After 24 hr cells were treated for 48 hr with DMSO or selumetinib (MEKi) before they were analyzed for cell number and on a Western blot for the indicated proteins. All quantitative data indicate mean values ±SEM. $^{**}p < 0.01$. (E) Fold change in PAX3 and SKI expression in 5 patients during vemurafenib (#3, #4, #24) or dabrafenib/trametinib (#9, #25) treatment and at time of progression. Data show box plots indicating the upper/lower quartile and the median with whiskers from min to max values $^{*}p < 0.05$; $^{**}p < 0.01$. (F) Fold change in *MITF* expression in the indicated patients. Data indicate the mean ±SEM. (G) Fold change in MITF and DUSP6 expression in the indicated patients. Data show box plots indicating the upper/lower quartile and the median with whiskers from min to max values. A phospho-ERK immunohistochemistry of 3 patients before treatment and at the time of progression is shown; scale bars: 100 µm. (H) Fold change in the expression of the indicated genes. Raw microarray data was obtained from Gene Expression Omnibus (GEO, accession GSE50509 (Patient 5 and 10) and GSE61992 (Patient 1)) and re-analyzed. (I, J) Publicly available gene expression data sets GSE50509 (I, 21 patients, 21 pre-treatment, 29 progressed samples) and GSE61992 (J, 9 patients, 9 pre-treatment, 9 progressed samples) were analyzed for PAX3 and MITF expression.

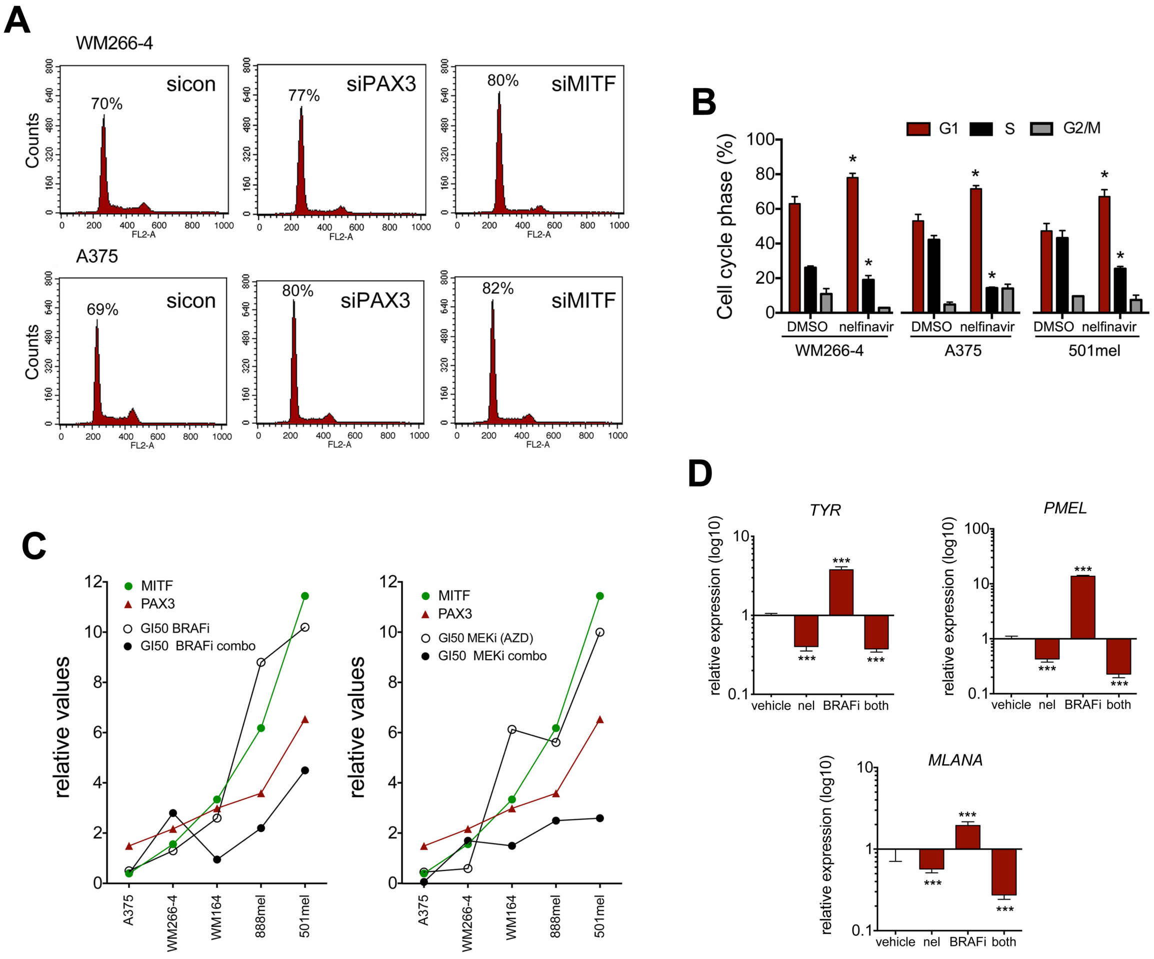

Figure S6 (related to Figure 6): Nelfinavir induces a G_1-arrest and its activity is related to MITF expression and function

(A) FACS analysis of WM266-4 and A375 cells treated with a control or PAX3 or MITF specific siRNA. Cells were analyzed 48 hr after the transfection. (B) Quantification of cell cycle distributions determined by FACS analysis of melanoma cells treated with DMSO or 10μM nelfinavir for 24 hr. Data indicate the mean ±SEM. *p < 0.05. (C) MITF and PAX3 expression was analyzed by quantitative real-time and the relative expression levels are shown. GI_{50} values for selumetinib (MEKi) or vemurafenib (BRAFi) and for the respective MAPKi in the presence of 10μM nelfinavir was determined and relative values are shown. (D) Real-time qPCR for *TYR*, *PMEL* and *MLANA* expression in A375 xenografts isolated from nude mice treated with vehicle, nelfinavir (25 mg/kg/qd) or PLX4720 (BRAFi; 25 mg/kg/qd) either alone or in combination for 21 consecutive days. Mean expression ±SEM relative to vehicle control is shown, ***p < 0.001.

Table S2 (related to Figure 7): Patient Characteristics corresponding to short-term cultures

Cell culture	Treatment	Treatment Duration (days)	Mutation	Tumour Location
M121224	LGX818	115	$BRAF^{V600E}$ & $NRAS^{Q61K}$	Cutaneous
M130429	MEK162	57	$MITF^{E318K}$ & $NRAS^{Q61R}$	Bone
M130219	MEK162	21	$MITF^{E318K}$ & $NRAS^{Q61R}$	Cutaneous

Patients with metastatic melanoma harboring a *BRAF*^{V600E} or *NRAS*^{Q61R} mutation (confirmed by genotyping) were enrolled on clinical trials for treatment with LGX818 (BRAFi) or MEK162 (MEKi). Mutation, treatment and duration until melanoma cells were isolated from the indicated tumor location are indicated.

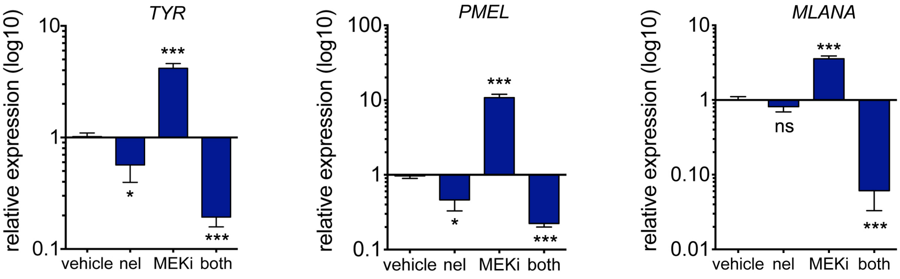

Figure S7 (related to Figure 8): Melanoma differentiation gene expression in nelfinavir treated tumors

Real-time qPCR for *TYR*, *PMEL* and *MLANA* expression in M249-R4 xenografts isolated from nude mice treated with vehicle, nelfinavir (25 mg/kg/qd) or selumetinib (MEKi; 25 mg/kg/qd) either alone or in combination for 21 consecutive days. Mean expression ±SEM relative to vehicle control is shown, *p < 0.05; ***p < 0.001.

Supplemental Experimental Procedures

Cell lines

A375 and WM266-4 cells were bought from the American Type Culture Collection and 501mel and 888mel cells were a gift from Steve Rosenberg (NCI, MD); all were obtained in 2008. Additional cell lines in the panel were a gift from Meenhard Herlyn (Wistar Institute) and Adam Hurlstone (University of Manchester). All cell lines were authenticated in house by short tandem repeat profiling before and during the study; the last authentication was carried out in 2014. These cell lines were grown in DMEM/10% FCS (PAA, Yeovil, UK).

In vivo xenograft studies

All animal procedures involving animals were ethically approved and carried out under license in accordance with the UK Home Office Animals (Scientific Procedures) Act (1986) and guidelines of the Committee of the National Cancer Research Institute for the Welfare and Use of Animals in Cancer Research (Workman et al., 2010). Nude mice were injected sc with 4×10^6 A375 cells (in PBS) or M249-R4 cells (in matrigel). When animals had developed melanoma nodules of about 100 mm^3, drug administration was initiated. Treatment was by oral gavage once daily with vehicle (5% DMSO, 95% water), or the respective drugs as indicated. After the indicated number of days tumors were isolated and analyzed as described. Zebrafish (*Danio rerio*) were raised and maintained at the University of Manchester Biological Services Unit. Zebrafish xenografts were generated by injection of approximately 1000 melanoma cells into the space surrounding the heart of embryos 48 hours post-fertilization. Subsequently, groups of six larvae per condition, randomly assigned were treated with either PD184352 (20nM) or nelfinavir (5µM) alone or in combination or the vehicle DMSO. The drug was added to the fish medium, and larvae were grown at 34°C ambient temperature in chorion water. Before drug addition (day 1) and 3 days after drug addition, anesthetized larvae were imaged using a Leica SP5 confocal microscope. Images were processed using Volocity software (Perkin Elmer, Cambridge, UK.) and GFP-green was false colored as red.

Immunohistochemistry and immunofluorescence

5 µm sections of paraffin-embedded tumor were de-waxed, rehydrated and subjected to antigen retrieval in citrate buffer (pH 6.0) for 30 min at 98 °C. The slides were blocked in 10% goat serum and incubated in primary antibody phospho-ERK (Cell Signaling Technology, Danvers, MA, USA) or total ERK2 ((C-14) Santa Cruz Biotechnology, Santa Cruz, CA, USA) overnight. After washing, the sections were incubated with biotinylated

secondary antibody for 1hr. Staining was visualized by applying a streptavidin–biotin complex followed by 3,3-diaminobenzidine (DAB) (Sigma, St Louis, MO, USA). Sections were counterstained with hematoxylin. Control sections were incubated with rabbit IgG. For immunofluorescence analysis tumor cryo-sections were permeabilised in a solution of 0.1 % Trition-X100 and 1 % saponin in PBS for 15 min. Sections were blocked in 10 % BSA at 37°C for 30 min and incubated overnight at 4°C with primary MITF antibody ((C5) Neomarkers, Lab Vision, Runcorn, UK) in 10 % BSA PBS. Stained sections were washed in PBS and then incubated with Cy3-labelled secondary antibody for 2 hr at RT and mounted using DAPi containing vectashield. For cell staining, cells were fixed with 4 % formaldehyde in PBS for 15 min before permeabilisation with 0.1 % Triton-X100 and the protocolled followed as described above using the indicated antibodies.

RNA analysis and qPCR primers

RNA from cell lines or frozen tumor tissue was isolated with TRIZOL® as described previously (Smith et al., 2013; Smith et al., 2014). Selected genes were amplified using SYBR green (Qiagen, Valencia, CA, USA) or TaqMan probes. Patient samples were pre-amplified using the TaqMan PreAmp Master Mix Kit (PN4384267, Applied Biosystems, Carlsbad, CA) according to the manufacturer's instructions. TaqMan qPCR was carried out with TaqMan Gene Expression Master Mix (PN4369016, Applied Biosystems, Carlsbad, CA). Primer sequences for SYBR green qPCR were: PAX3: AGGATGCGGCTGAT-GGAACTCACTG, CCAGGATGATGCGGCCGGGCCCGGG; SKI: CAGGAGCTGGAGTT-CCTACG, GTGACTCGTTGGCCTCTTTC; M-MITF: CCGTCTCTCACTGGATTGGT, TACT-TGGTGGGGTTTTCGAG; MLANA/MART-1: TTGGCACTCAATGTCCTTA, GGGAACCAC-AGGTTCACAGT; TYROSINASE: CTGGAAGGATTTGCTAGTCCAC, CCTGTACCTGGGA-CATTGTTC; B-ACTIN: GCAAGCAGGAGTATGACGAG, CAAATAAAGCCATGCCAATC; CXCL1: AACCGAAGTCATAGCCACAC, GTTGGATTTGTCACTGTTCAG; CXCL2: GGGC-AGAAAGCTTGTCTCAA, GCTTCCTCCTTCCTTCTGGT; EGF: AAGGTACTCTCGCA-GGAAATGG, ACATACTCTCTCTTGCCTTGACC; OPN: AGTTTCGCAGACCTGACAT-CCAGT, TTCATAACTGTCCTTCCCACGGCT; CCL2: GATCTCAGTGCAGAGGCTCG, TGCTTGTCCAGGTGGTCCAT; SNAIL: GAGCATTTGCAGACAGGTCA, TCCTCATGTT-TGTGCAGGAG; ZEB2: GTGTCACTGCGCTGAAGGTA, GTGTCACTGCGCTGAAAGGF or TaqMan qPCR the individual probes were: ACTB:Hs99999903; PAX3: Hs00240950; MITF: Hs00165156.

Cell culture reagents, transfections and siRNAs

PD184352 (MEKi) was from Axon Medchem, (Groningen, The Netherlands), and vemurafenib (BRAFi), selumetinib (MEKi) and trametinib (MEKi), RAF265 (a pan RAF inhibitor) were from Selleck Chemicals (Newmarket, UK). Nelfinavir was from Sigma-Aldrich (Gillingham, UK). Cells were transfected with plasmid DNA using Lipofectamine (Life technologies, USA) and with siRNAs using Interferin (Polyplus, Illkirch, France) following the manufactures instructions. Over-expression experiments used pEFMITFD, pEFPAX3, pRSV-SKI and pRK5F-SMAD2 expression plasmids. siRNAs were from Dharmacon/Thermo Fisher or Ambion and the sequences were: si-control AAUAUAAUCACUAUCAGGUGC; MITF: #1 GAACGAAGAAGAAGAUUUAUUU, #2 AAAGCAGUACCUUUCUACCAC, #3 GACCUAACCUGUACAACAAUU; PAX3: #1 CCGAGACAAAUUACUCAAGGA; SMAD4: #1 GGUCUUUGAUUUGCGUCAG, #2 CCCACAACCUUUAGACUGA, #3 CGAAUUGAAAGUUUGGUA; SKI: ON-TARGET plus SMART pool: GCGACGAGCUCCACAUCUA, GAAAGAGGCCAACGAGUCA, CCACGGAGGCCAAGCGUAA, UGAAGGAGAAAUUCGACUA.

Drug Screen

WM266-4 cells were plated in 96 well microplates (Becton Dickinson 353219) at a density of 500 cells per well. 48 hr later cells were treated with the ENZO Life Sciences FDA approved drug library BML-2841-0100 (at 10μM). 24 hr later cells were fixed with 4% PFA-PBS, and incubated with antibodies against either MITF (clone C5, Neomarkers/Lab Vision) or PAX3 (Developmental Studies Hybridoma Bank, UIOWA) in 2% BSA-PBS. Antibodies were detected using Cy3-coupled secondary antibodies (Jackson ImmunoResearch, UK). Nuclei were identified by staining with Hoechst 33342 (Sigma). siRNA mediated knock down of MITF and PAX3 (decrease) as well as 6h 0.5 μM protease inhibitor ALLN (Santa Cruz, USA) treatment (increase) were used as control. Cy3 signal intensity was quantified on a BD Pathway 435 system using BD AttoVision™ v1.7 software in the form of a 12 x 12 montage using a 20x (0.75NA) objective in non-confocal mode. Image analysis was performed by selecting regions of interest (ROI) based on Hoechst 33342 (Sigma) staining. The intensity of the Cy3 signal was measured within ROI, an average Cy3-signal intensity was calculated per well using BD Image data explorer (IDE) software, and relative signal values were calculated taking background values into account. Standard deviations for each well were calculated to determine the z-score. Cell survival was measured using ROI values. And survival in the DMSO control was set 100% Results are based on at least two independent repeats for each plate in each screen.

Immunoprecipitation and Chromatin Immunoprecipitation

For immunoprecpitation, SKI was precipitated from 1mg of total protein using 2μg of SKI antibody. Chromatin immunoprecipitation assays using control IgG (Santa Cruz) or antibodies specific for SKI (H-329) were performed were performed 24 hr after treatment with 10μM Nelfinavir, 1μM selumetininb or both and carried out as described previously (Wellbrock et al, 2008). Primers for the PAX3 promoter were fwd TGGGGCTGTCTCTCTCAGTT; rev TTACCCAAAGCTTGGTCAGG and the negative control PAX3 intron 2 fwd ACCTCCAAACACCCTCCTCT; rev CTGCAACTTGTGCTTTTCCA as used in (Yang et al, 2008).

Supplemental References

Smith, M. P., Sanchez-Laorden, B., O'Brien, K., Brunton, H., Ferguson, J., Young, H., Dhomen, N., Flaherty, K. T., Frederick, D. T., Cooper, Z. A., et al. (2014). The immune microenvironment confers resistance to MAPK pathway inhibitors through macrophage-derived TNFalpha. Cancer discovery *4*, 1214-1229.

Workman, P., Aboagye, E. O., Balkwill, F., Balmain, A., Bruder, G., Chaplin, D. J., Double, J. A., Everitt, J., Farningham, D. A., Glennie, M. J., et al. (2010). Guidelines for the welfare and use of animals in cancer research. British journal of cancer *102*, 1555-1577.
